# Efficacy and safety of multiple external therapies in patients with insomnia: a systematic review and network meta-analysis

**DOI:** 10.3389/fneur.2024.1297767

**Published:** 2024-07-05

**Authors:** Zhen Wang, Hui Xu, Zheng Wang, Hang Zhou, Lijuan Zhang, Yu Wang, Miaoxiu Li, Yunfeng Zhou

**Affiliations:** ^1^College of Acupuncture and Massage, Henan University of Chinese Medicine, Zhengzhou, China; ^2^Department of Tuina, The Third Affiliated Hospital of Henan University of Chinese Medicine, Zhengzhou, China; ^3^Central Hospital of Jiaozuo, Jiaozuo, China; ^4^College of Computer Science, Xidian University, Xian, China; ^5^College of Acupuncture and Massage, Shanghai University of Chinese Medicine, Shanghai, China

**Keywords:** insomnia, external treatment, network meta-analysis, neurotransmitter, psychological state

## Abstract

**Background:**

The annual incidence of insomnia continues to increase owing to changes in lifestyle habits, increased work pressure, and increased environmental pollution. In recent years, an increasing number of external therapies have been proven effective in treating insomnia and have been widely used. However, the relative benefits and harms of external therapies remain uncertain, and an optimal treatment strategy has not yet been determined.

**Objectives:**

A network meta-analysis was performed to evaluate and compare the efficacy and safety of multiple external therapies for patients with insomnia.

**Methods:**

Eight electronic databases were comprehensively searched from their inception to June 2023 for relevant literature. We also searched the grey literature and reviewed the reference lists of related systematic reviews. Two independent reviewers performed the study selection, data extraction, and bias assessment of the included randomized controlled trials (RCTs) using the Cochrane Reviewers’ Handbook, and a network meta-analysis was conducted using Stata and RevMan software.

**Results:**

In total, 14,826 studies were identified. Of these, 83 studies, including 9 external therapies and 6,100 patients, were deemed eligible for the present network meta-analysis. Except for the SL outcome, each external therapy was better than conventional medicine and the sham intervention (SI) in improving sleep quality. In terms of improving the psychological state indices of insomnia patients, each external therapy was superior to the SI; each external therapy had a better effect on the regulation of monoamine neurotransmitters. Tuina may be the most effective intervention in improving the total effective rate, Pittsburgh sleep quality index score, and SL. Repetitive transcranial magnetic stimulation (rTMS) perhaps resulted in the best improvement in total sleep time and awakening time (surface under the cumulative ranking curve [SUCRA] = 78.3 and 75.4%, respectively); and moxibustion (MB) and hyperbaric oxygen (HBO) were the most effective in reducing Self-rating Anxiety Scale and Self-rating Depression Scale scores. In terms of improving the monoamine neurotransmitters 5-hydroxytryptamine, norepinephrine, and dopamine, the best interventions were acupoint catgut embedding, electroacupuncture, and Tuina (SUCRA = 82.0, 69.9 and 90.3%, respectively). Safety results showed that the three safest interventions were the SI, Tuina, and foot bath. No serious adverse events were reported across the studies, and the most common minor adverse events included drowsiness, pain, excessive thirst, and hematoma.

**Conclusion:**

Both Tuina and rTMS have significant effects on improving sleep quality, but the safety of rTMS is low. Therefore, Tuina can be recommended as the first line of treatment to improve sleep quality. If a patient’s anxiety and depression symptoms are evident, MB or HBO can be selected for treatment based on the actual situation. External therapy to improve sleep quality may be related to the regulation of monoamine neurotransmitters, which may be a potential mechanism of action.

**Systematic Review Registration:**

https://www.crd.york.ac.uk/PROSPERO/display_record.php?RecordID=440882.

## 1 Introduction

Insomnia is a sleep disorder characterized by difficulty falling asleep, easy awakening, and early waking, and is a common clinical condition (1, 2). With the increasing work pressure and the accelerated pace of life, the incidence of insomnia is increasing annually and the disease can last for more than 10 years in severe cases (3, 4). A chronic lack of sleep can lead to emotional and mental problems such as anxiety and depression (5). Recent studies have shown that the severity of anxiety and depression positively correlates with the occurrence of insomnia (6, 7). The mechanisms of insomnia are complex and related to a variety of neurotransmitters (8). It is widely recognized that abnormal levels or reduced function of neurotransmitters underly the pathophysiology of insomnia (9, 10). Drug treatments for insomnia are commonly used and have the advantages of being easy to take, fast-acting, and long-lasting. However, the incidence of adverse effects is high, and it is more difficult for patients to adhere to treatment (11–13). Therefore, it is necessary to explore alternative therapies with significant, curative, stable, and safe effects.

Attention to non-pharmacological therapies is increasing because of concerns about the safety of pharmaceutical treatments. Non-pharmacological therapies mainly include external therapy, exercise and cognitive behavioral therapy. Because exercise and cognitive behavioral therapy make it difficult for patients to adhere, more and more patients choose external therapy for treatment. External therapy is a method of treatment from outside the body using instruments, needles and human fingers, which is greener and safer than drug therapy. Relevant studies (14, 15) have found that external therapies for insomnia have the advantages of significant efficacy, good safety profiles, and few adverse effects, and have become a popular topic in recent years. Several guidelines and consensuses (16, 17) list external treatments as the recommended intervention for the clinical treatment of insomnia. External therapies include modern medical and traditional Chinese medicine (TCM) treatments (18). Modern medical treatments include repetitive transcranial magnetic stimulation (rTMS), hyperbaric oxygen, electroencephalography (EEG) biofeedback, and laser therapy, whereas TCM treatments primarily include acupuncture, Tiuna, auricular stimulation, and moxibustion (19).

Different types of external therapies have different effects and advantages. Several traditional meta-analysis (20–22) have proven that external treatment of insomnia has advantages, but they are more focused on the comparisons of a single external therapy with drugs or another external therapy and did not compare multiple external therapies simultaneously. As the number of alternative treatment options increases, comparative effectiveness and safety studies are necessary (23). To date, no meta-analyses have comprehensively compared and evaluated the efficacy and safety of multiple types of external therapies. Thus, it is unknown which intervention measures have the best effects. Moreover, most systematic reviews focus only on the changes in insomnia-related indicators, mostly using subjective scale evaluations, and fail to explore the changes in the psychological state and neurotransmitter levels of insomnia patients under the intervention of external therapy. Therefore, a network meta-analysis was performed to simultaneously analyze both direct and indirect evidence from different studies, estimate the relative effectiveness and safety of all interventions, and rank the order of the interventions (24, 25). The aim of this study was to systematically evaluate the effects of external therapies on the psychological state and neurotransmitters of patients with insomnia based on the evaluation of insomnia-related indicators.

## 2 Methods

### 2.1 Inclusion criteria

#### 2.1.1 Participants

All studies met the recognized diagnostic criteria for insomnia (26–29), regardless of age, sex, or race.

#### 2.1.2 Interventions

The trial group received external therapy alone, in which drug involvement was excluded, including acupoint catgut embedding (ACE), rTMS, tuina, hyperbaric oxygen (HBO), electroacupuncture (EA), moxibustion (MB), foot bath (FB), manual acupuncture (MA), and auricular stimulation (AS).

#### 2.1.3 Controls

The control group included patients receiving conventional medicine (CM), sham intervention (SI), or any of the external therapies mentioned above (e.g., HBO vs. MB). To reduce heterogeneity, only benzodiazepines were used in CM.

#### 2.1.4 Outcomes

##### 2.1.4.1 Sleep quality

The following outcome measures were used to assess sleep quality: (1) The total effective rate, referring to efficacy standards formulated by the State Administration of Traditional Chinese Medicine and American Psychiatric Association (27, 28). The total effective rate is calculated as follows: [(Cure + marked effect + effective) number of cases ÷ total number of cases] × 100%; (2) Pittsburgh sleep quality index (PSQI); and (3) Data from polysomnography (PSG) including total sleep time (TST), sleep onset latency (SOL), and awakening time (AT).

##### 2.1.4.2 Psychological state

The Self-rating Anxiety Scale (SAS) and Self-rating Depression Scale (SDS) were used to assess the patients’ psychological status.

##### 2.1.4.3 Neurotransmitter level

The levels of 5-hydroxytryptamine (5-HT), dopamine (DA), and norepinephrine (NE) were analyzed.

##### 2.1.4.4 Adverse reaction

The incidence of adverse reactions was used to evaluate the safety of the intervention.

All RCTs that contained at least one outcome indicator were eligible for inclusion in the network meta-analysis (NMA).

#### 2.1.5 Study design and registration

This study followed the Preferred Reporting Items for Systematic Reviews and Meta-analysis network meta-analysis (PRISMA-NMA) guidelines (30) and was registered with PROSPERO (registration number: CRD42023440882).

### 2.2 Exclusion criteria

Exclusion criteria included: (1) Non-RCTs; (2) repeated publications; (3) inconsistent interventions; (4) no reference or homemade diagnostic criteria; (5) inability to obtain full text and outcomes; and (6) serious complications.

### 2.3 Information sources

The Cochrane Library, Embase, PubMed, Web of Science, Chinese Biomedical Databases, VIP, Chinese National Knowledge Infrastructure, and Wanfang databases were searched for relevant studies. We also searched the grey literature and reviewed the reference lists of the included studies and related systematic reviews. There were no restrictions regarding language, study type, date of publication, or status of publication. The retrieval strategy used a combination of subject headings and free words, and the databases were searched from inception until June 30, 2023. Each database search strategy is shown in Supplementary Figure S1 and Supplementary Table S1.

### 2.4 Study selection

Two researchers independently screened the studies based on the inclusion criteria. After extracting the data, they crosschecked each other’s results. Disagreements were resolved through consultation with a third party. EndNote software was used to check for duplicate publications. The investigators then screened the titles and abstracts of each study and excluded studies that did not meet the inclusion criteria. Subsequently, the investigators read the full texts of the remaining studies to decide whether to include them. If the literature was incomplete, the authors of the original studies were contacted to obtain detailed data.

### 2.5 Data collection

Two reviewers independently extracted the data from each eligible RCT using a standardized form. Disagreements were resolved through consultation with a third party. The extracted data included study characteristics (author, country, and publication date), patient characteristics (sample size, disease duration, sex, and age), intervention measures, treatment course, and outcome indicators.

### 2.6 Risk of bias assessment

The risk of bias in the literature was evaluated by two independent investigators using the risk of bias assessment tool in the Cochrane Reviewers Handbook (31). The following seven aspects were evaluated to determine the risk of bias: random sequence generation, allocation concealment, implementation of a blind method for patients and trial personnel, implementation of a blind method for outcome assessors, incomplete result data, selective reporting, and other biases (such as potential bias related to special study designs and false statements). Eventually, a judgment on “low risk,” “high risk,” and “unclear risk” in the selected literatures was made.

### 2.7 Data synthesis and analysis

All outcome indicators were analyzed using random- or fixed-effects models based on the level of heterogeneity. The *p* values of the chi-square test and the I^2^ index in the heterogeneity test were used to indicate the level of statistical heterogeneity. When the level of heterogeneity was low, the data was analyzed using the fixed effects model (*p* ≥ 0.1 and *I*^2^ < 50%); otherwise, the random effects model (*p* < 0.1 or *I*^2^ > 50%) was used (32, 33). Relative risk (RR) was used as the effect size for dichotomous variables and the standardized mean difference (SMD) was used as the effect size for continuous variables to calculate the 95% confidence interval (CI).

Based on the Bayesian model, Stata software (version 16.0) was used for network meta-analysis. The data was preprocessed using the network group command, and an evidence network diagram for each indicator was drawn. The curative effect of the indicators was sorted to obtain the surface under the cumulative ranking curve (SUCRA), and probability sorting was plotted. The dots in the evidence network diagram represent an intervention; the larger the area, the greater the number of patients with the intervention. The line connecting the two dots indicates a direct comparison between the two interventions, and the thickness of the line represents the number of included studies (34, 35). SUCRA is expressed as a percentage. A larger percentage indicates that the intervention has the highest probability of becoming the preferred option, and a value of zero indicates that the intervention may be completely ineffective (36, 37). When a closed loop exists, the node-splitting method is used to check for inconsistencies. When the number of studies on the outcome indicator was >10, a funnel plot was drawn to determine the possibility of a small sample effect. To test the robustness of the main findings, some factors that might influence the level of precision of the main outcome were removed, a sensitivity analysis was performed, and the quality of the literature was evaluated using Review Manager 5.4 software.

## 3 Results

**Figure 1:**
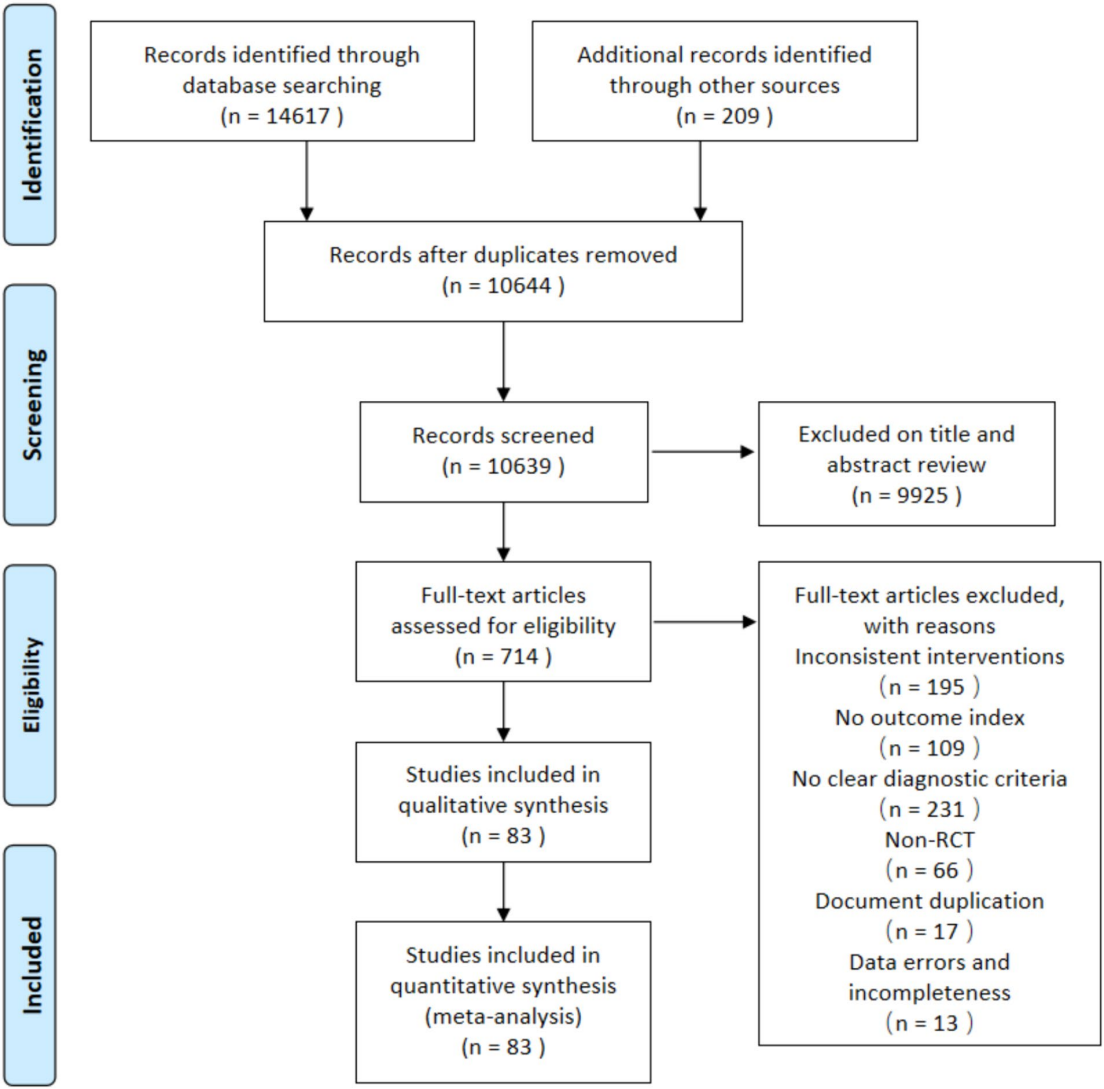
Literature search process.

A total of 14,826 articles were retrieved from the initial search, of which 14,617 were from databases and 209 were from other sources. Among the literatures retrieved in the database, 4,239 were from CNKI, 2611 VIP, 3846 Wanfang, 2,973 CBM, 97 Embase, 205 Cochrane Library, 382 PubMed, and 264 Web of Science. Then, 714 were moved to full-text screening after title and abstract screening. Finally, we included 83 RCTs (38–120) in our meta-analysis, of which eight studies (38, 41, 54, 73, 77, 95, 112, 120) were three-arm trials and the remaining studies were two-arm trials. The screening flowchart is shown in Figure 1.

### 3.1 Study characteristics

Supplementary Table S2 summarizes the characteristics of the 83 RCTs, with sample sizes ranging from 32 to 153, involving 6,100 participants with a mean age of 18–68 years; nine external therapies, including ACE, rTMS, Tuina, HBO, EA, MB, FB, MA, and AS. Descriptions of each intervention are presented in Supplementary Table S3.

### 3.2 Risk of bias

**Figure 2:**
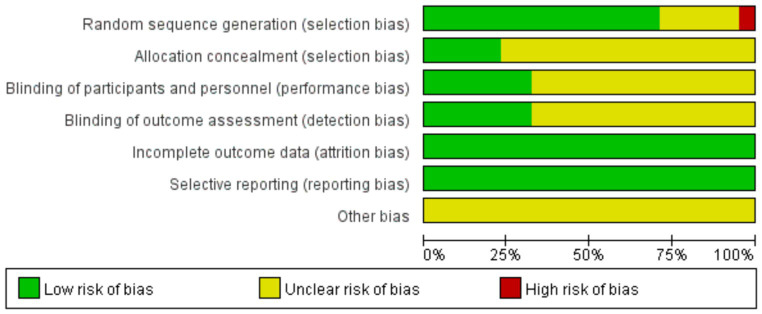
Literature bias evaluation results.

Of the 83 included RCTs, 18 (57, 58, 73–77, 82, 98, 104–109, 113, 114, 119) were published in English, and the remainder were published in Chinese. The studies had comparable general information between the control and trial groups. Seventy-nine studies reported specific schemes for random sequence generation, with 41 (38, 42, 44, 46, 47, 50, 51, 53, 56, 60, 62, 64–66, 68, 70, 71, 73, 74, 80–86, 88, 90, 91, 96, 98–103, 110, 112, 114, 119, 120) using the random number table method and 17 (40, 43, 61, 72, 75–78, 89, 97, 104–109, 113) using the envelope method. One study (45) was rated as low risk using the coin-toss method for random allocation, two (39, 63) were rated as high risk according to the order of consultation, two (67, 87) were randomized according to different treatments, and the remaining 20 studies mentioned randomization only. Twenty-seven studies mentioned the implementation of blinding: 21 (40, 43, 59, 61, 64, 72–76, 78, 97, 99, 104, 106–109, 112, 113, 115) were single-blinded, six (58, 77, 89, 92, 105, 114) were double-blinded, 19 (40, 43, 61, 64, 72, 75–78, 89, 97, 104–109, 113, 114) used allocation concealment rated as low risk, and the remaining studies did not mention blinding or allocation concealment. All 83 studies reported on the outcome indicators used in this study, and the studies did not identify falsified or incomplete data, with incomplete reporting and early discontinuation of trials rated as low risk. No other biases were mentioned in any of the studies. The results are shown in Figure 2, and a summary of the risk of bias is shown in Supplementary Figure S2.

### 3.3 Network meta-analysis

The results of the heterogeneity test showed high heterogeneity for all outcome indicators (*p* < 0.05, I^2^ > 50%). Therefore, a random-effects model was used for all the meta-analyses in this study. The heterogeneity results are presented in Supplementary Table S4. Except for 5-HT, NE, and DA, the evidence network diagrams of the outcome indicators were closed loops. The node-splitting method showed good consistency with no heterogeneity emerging between the studies (*p* > 0.05). The results of the node-splitting tests are listed in Supplementary Tables S5–S12.

#### 3.3.1 Sleep quality

##### 3.3.1.1 Total effective rate

**Figure 3:**
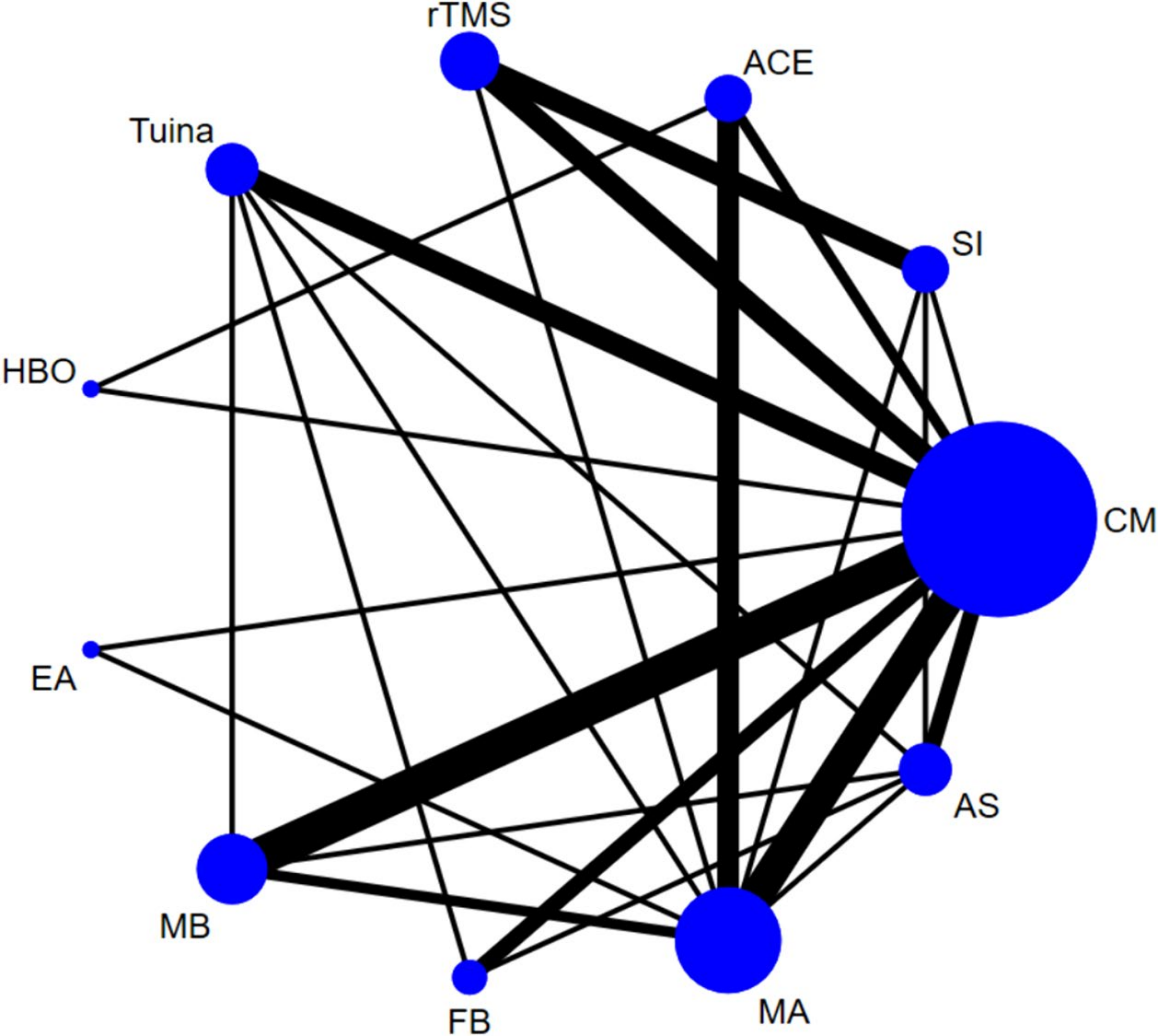
Network diagram of the total effective rate.

**Figure 4:**
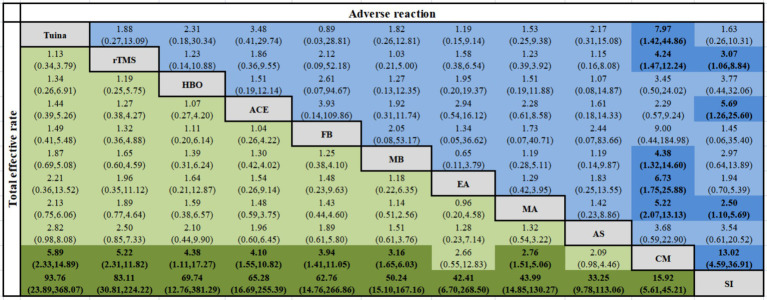
Network meta-analysis of the total effective rate and adverse reaction rate [RR (95% CI)].

Forty-four studies (38–43, 45, 46, 48, 50, 51, 54–56, 62–65, 68, 71, 78–81, 83–88, 90, 92, 94–96, 99, 101–103, 110, 113, 115, 116, 119, 120) reported the total effective rate, involving 3,223 participants and 11 interventions. Thus, 55 two-by-two comparisons were formed, and the evidence network was generally centered on the CM, thereby forming 19 closed loops (see Figure 3). Compared to SI, all external therapies and CM showed a better effect on the total effective rate (*p* < 0.05). Except for EA and AS, external therapies were superior to CM in improving the total effective rate (*p* < 0.05). There were no significant differences in most comparisons between the external treatments (*p* > 0.05) (Figure 4).

##### 3.3.1.2 PSQI

**Figure 5:**
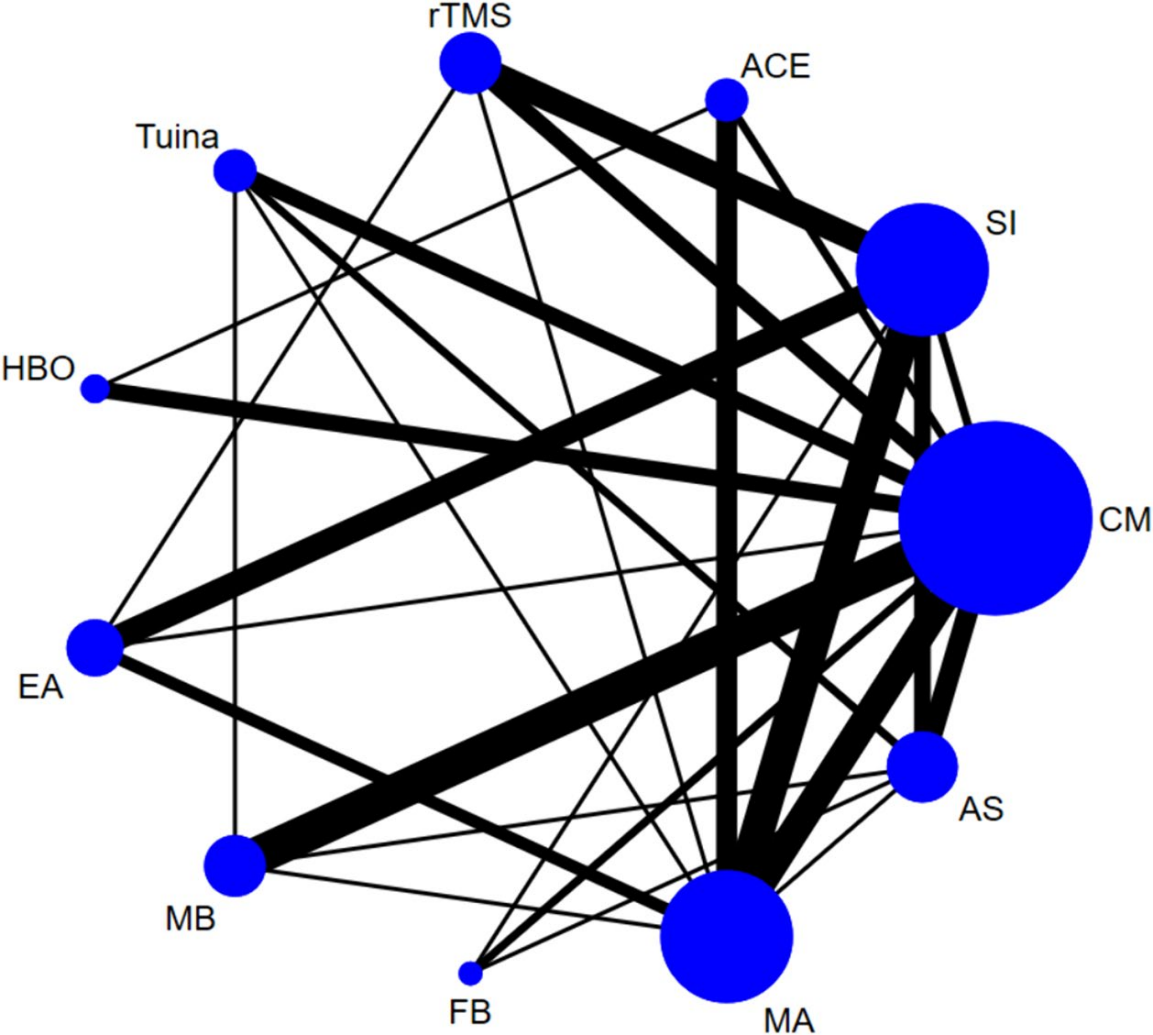
Network diagram of Pittsburgh sleep quality index scores.

Seventy studies (38–43, 45, 47–50, 53–69, 71–86, 88–91, 93, 94, 97–104, 106–108, 110, 112–120) reported the PSQI score, involving 5,224 participants and 11 interventions; thus, 55 two-by-two comparisons were formed, with an overall network of evidence centered on CM, thereby forming 24 closed loops (see Figure 5). The results of the NMA showed that Tuina, rTMS, ACE, and MB significantly reduced the PSQI score compared with AS, and Tuina, rTMS, ACE, HBO, FB, MB, and MA were better than CM. Compared with SI, all external treatments and CM showed a better effect on decreasing the PSQI total scores. All the above-mentioned differences were statistically significant (*p* < 0.05), as shown in Supplementary Figure S12.

##### 3.3.1.3 TST

Twenty-three studies (46, 48, 51, 52, 57, 60, 61, 66, 67, 73–77, 81, 101, 104–106, 109, 110, 112, 118) reported the TST, involving 1,814 participants and 9 interventions. Thus, 36 two-by-two comparisons were formed, and the evidence network was generally centered on the SI, leading to eight closed loops (see Supplementary Figure S3). Compared with SIs, rTMS (MD = 51.23, 95% CI [15.57, 86.89]), MA (MD = 36.43, 95% CI [13.49, 59.38]), and EA (MD = 28.55, 95% CI [3.57, 53.54]) significantly improved the TST (*p* < 0.05), as shown in Supplementary Figure S12.

##### 3.3.1.4 SL

Twenty-one studies (46, 48, 51–53, 56, 57, 60, 61, 66, 67, 73, 74, 76, 81, 101, 104–106, 110, 118) reported SL, involving 1,631 participants and 9 interventions. Thus, 36 two-by-two comparisons were formed with an overall network of evidence centered on CM, thereby forming six closed loops (see Supplementary Figure S4). Compared with SI, rTMS (MD = −12.48, 95% CI [−22.95, −2.01]) and MA (MD = −11.34, 95% CI [−20.06, −2.62]) significantly reduced SL. rTMS (MD = −10.88, 95% CI [−21.56, −0.19]) was a superior intervention to CM. All the abovementioned differences were statistically significant (*p* < 0.05), as shown in Supplementary Figure 13.

##### 3.3.1.5 AT

Fourteen studies (6, 48, 51–53, 60, 61, 66, 67, 75, 77, 81, 101, 112) reported SL, involving 1,089 participants and 9 interventions. Thus, 36 two-by-two comparisons were formed, and the evidence network was generally centered on CM, thereby forming five closed loops (see Supplementary Figure S5). The results of the NMA showed that rTMS was a better intervention than CM and SI [MD = −1.31, 95% CI (−2.42, −0.20); MD = −2.01, 95% CI (−3.56, −0.46)], respectively; *p* < 0.05) (Supplementary Figure S13).

#### 3.3.2 Psychological state

##### 3.3.2.1 SAS

Eighteen studies (42, 45, 47, 56, 63, 70, 75, 82, 83, 87, 93, 96, 101, 103, 108, 109, 115, 116) reported SL, involving 1,462 participants and 11 interventions. Thus, 55 two-by-two comparisons were formed, with an overall network of evidence centered on CM, thereby forming only one closed loop (see Supplementary Figure S6). The results of the NMA showed that MB, HBO, MA, and rTMS significantly reduced the SAS score compared to SI. MA (MD = −5.52, 95% CI [−10.96, −0.08]) was a better intervention than CM. Compared with Tuina, MB (MD = -14.68, 95% CI [−27.90, −1.47]) was more effective at reducing SAS scores. All the abovementioned differences were statistically significant (*p* < 0.05), as shown in Supplementary Figure S14.

##### 3.3.2.2 SDS

Nineteen studies (42, 45, 47, 56, 63, 70, 73, 75, 77, 82, 87, 89, 99, 101, 103, 108, 109, 115, 116) reported SL, involving 1,555 participants and 10 interventions. Thus, 45 two-by-two comparisons were formed, and the evidence network was generally centered on the MA, thereby forming two closed loops (see Supplementary Figure S7). Compared to SI, MB, HBO, MA, and EA significantly reduced the SDS score. HBO was a better intervention than CM (MD = −17.39, 95% CI [−32.19, −2.59]) and Tuina (MD = −23.02, 95% CI [−43.73, −2.31]). All the abovementioned differences were statistically significant (*p* < 0.05), as shown in Supplementary Figure S14.

#### 3.3.3 Neurotransmitters

##### 3.3.3.1 5-HT

Twenty studies (43, 44, 46, 51, 55, 59, 65, 66, 71, 81, 84, 88, 92, 94, 96, 100, 102, 107, 111, 117) reported 5-HT as an outcome measure, involving 1,339 participants and 10 interventions. Thus, 45 two-by-two comparisons were performed, with an overall network of evidence centered on CM (Supplementary Figure S8). The results of the NMA demonstrated that with the exception of CM, most of the external therapies showed a better effect on 5-HT levels than SI (*p* < 0.05). Compared to CM, ACE, rTMS, Tuina, MA, and MB significantly reduced 5-HT levels (p < 0.05). No significant differences were found among the different external treatments (*p* > 0.05), as shown in Supplementary Figure S15.

##### 3.3.3.2 DA

Nine studies (44, 46, 51, 55, 59, 72, 94, 98, 117) reported DA as an outcome measure, involving 572 participants and eight interventions. Thus, 28 two-by-two comparisons were made, with an overall network of evidence centered on the CM and SI (see Supplementary Figure S9). The results of the NMA showed that rTMS, Tuina, EA, MA, and FB significantly improved DA levels compared with SI and CM. Compared with FB and AS, rTMS, Tuina, EA, and MA were more effective in increasing DA concentrations. rTMS, Tuina and EA were superior in performance to MA. All the abovementioned differences were statistically significant (*p* < 0.05; Supplementary Figure S15).

##### 3.3.3.3 NE

Ten studies (44, 51, 55, 56, 65, 71, 88, 98, 102, 111) reported NE outcome measures involving 679 participants and eight interventions; thus, 28 two-by-two comparisons were performed and the evidence network was generally centered on CM (see Supplementary Figure S10). Compared with SI, rTMS [MD = −18.55, 95% CI (−28.71, −8.38)] and AS [MD = −17.23, 95% CI (−31.26, −3.20)] significantly reduced the NE levels. rTMS and MA were more effective than CM in reducing NE levels. All the abovementioned differences were statistically significant (*p* < 0.05; Supplementary Figure S16).

#### 3.3.4 Adverse reactions

Of the 83 included articles, 46 reported adverse reactions. Among them, seven (43, 44, 64, 71, 72, 79, 104) reported no adverse events and 39 studies (38–42, 45–47, 49, 54, 56, 58, 59, 61, 68, 73–77, 80, 83–85, 89, 92, 97, 99–101, 103, 105, 107–109, 113–115) reported minor adverse reactions or no serious adverse reactions. Owing to the limited number of included studies and the broad range of definitions, adverse reactions could not be specifically subdivided, and only the total number of adverse reactions triggered directly by the interventions was analyzed. Specific adverse reactions are detailed in Supplementary Table S13. Forty-six studies involving 3,400 participants and 11 interventions reported adverse reactions. Thus, 55 two-by-two comparisons were formed, and the evidence network was generally centered on the MA, thereby forming 14 closed loops (see Supplementary Figure S11). The results of the NMA showed that Tuina, rTMS, MB, EA, and MA significantly improved adverse reactions when compared with CM. SI was safer than rTMS, ACE, MA, or CM. All the abovementioned differences were statistically significant (*p* < 0.05), as shown in Figure 4.

### 3.4 SUCRA probability ranking

#### 3.4.1 Sleep quality

##### 3.4.1.1 Total effective rate

Tuina was the most effective intervention in improving the total efficacy rate (82.5%), followed by rTMS (78.1%), HBO (67.6%), ACE (66.4%), FB (64.3%), EA (47.7%), MA (45.1%), AS (33.3%), CM (11.7%), SI (10.3%) and SI (0.0%).

##### 3.4.1.2 PSQI

Tuina was the most effective intervention (87.4%), followed by rTMS (83.2%), ACE (71.4%), HBO (64.3%), FB (62.5%), MB (59.3%), MA (47.5%), EA (40.7%), AS (20.9%), CM (12.8%), and SI (0.0%).

##### 3.4.1.3 TST

rTMS was the most effective therapy for improving the TST (78.3%), followed by Tuina (71.3%), MB (67.8%), MA (58.3%), AS (54.6%), EA (45.0%), HBO (38.5%), CM (29.0%), and SI (7.1%).

##### 3.4.1.4 SL

Tuina had the greatest effect on SL reduction (79.7%), followed by rTMS (69.0%), HBO (64.9%), MB (63.3%), MA (62.9%), AS (50.7%), CM (28.6%), EA (19.3%), and SI (11.6%).

##### 3.4.1.5 AT

rTMS was the most effective intervention for reducing AT (75.4%), followed by Tuina (69.0%), MB (65.5%), HBO (61.7%), AS (53.4%), EA (51.1%), MA (42.5%), CM (23.0%), and SI (8.3%).

#### 3.4.2 Psychological states

##### 3.4.2.1 SAS scores

Regarding SAS score reduction, MB was the most effective therapy (90.8%), followed by HBO (78.8%), MA (73.3%), ACE (63.5%), rTMS (59.3%), AS (52.8%), CM (40.5%), EA (29.7%), FB (25.9%), Tuina (23.1%), and SI (12.3%).

##### 3.4.2.2 SDS scores

HBO therapy had the best effect in terms of reducing SDS scores (93.7%), followed by MB (68.5%), MA (64.3%), ACE (63.2%), EA (58.1%), rTMS (46.2%), AS (45.6%), CM (31.9%), Tuina (16.8%), and SI (11.8%).

#### 3.4.3 Neurotransmitters

##### 3.4.3.1 5-HT

ACE had superior efficacy in increasing 5-HT levels (82.0%), followed by rTMS (75.9%), Tuina (70.5%), EA (61.8%), MA (57.9%), FB (51.3%), MB (50.2%), AS (35.1%), CM (14.4%), and SI (1.0%).

##### 3.4.3.2 DA

Tuina was the most effective intervention in improving DA levels (90.3%), followed by rTMS (86.9%), EA (79.9%), MA (57.2%), FB (39.7%), AS (24.2%), CM (17.8%), and SI (4.0%).

##### 3.4.3.3 NE

In terms of NE reduction, EA had the best effect (69.9%), followed by rTMS (67.4%), AS (61.9%), MA (57.8%), Tuina (55.8%), MB (55.5%), CM (20.2%), and SI (11.5%).

#### 3.4.4 Adverse reactions

SI was the safest intervention (88.7%), followed by Tuina (68.7%), FB (65.9%), EA (64.2%), MA (54.4%), MB (48.2%), rTMS (46.2%), AS (42.6%), HBO (41.1%), ACE (25.7%), and CM (4.2%). The SUCRA values and ranking results for each outcome indicator are shown in Table 1. Higher SUCRA values indicate more effective and safer interventions.

**Table 1:** Ranking of SUCRA probabilities for each outcome indicator.

Intervention	Total effective rate		PSQI		TST		SL		AT		SAS	
	SUCRA	RANK	SUCRA	RANK	SUCRA	RANK	SUCRA	RANK	SUCRA	RANK	SUCRA	RANK
Tuina	82.5	1	87.4	1	71.3	2	79.7	1	69.0	2	23.1	10
rTMS	78.1	2	83.2	2	78.3	1	69.0	2	75.4	1	59.3	5
HBO	67.6	3	64.3	4	38.5	7	64.9	3	61.7	4	78.8	2
ACE	66.4	4	71.4	3	-	-	-	-	-	-	63.5	4
FB	64.3	5	62.5	5	-	-	-	-	-	-	25.9	9
MB	53.3	6	59.3	6	67.8	3	63.3	4	65.5	3	90.8	1
EA	47.7	7	40.7	8	45.0	6	19.3	8	51.1	6	29.7	8
MA	45.1	8	47.5	7	58.3	4	62.9	5	42.5	7	73.3	3
AS	33.3	9	20.9	9	54.6	5	50.7	6	53.4	5	52.8	6
CM	11.7	10	12.8	10	29.0	8	28.6	7	23.0	8	40.5	7
SI	0.0	11	0.0	11	7.1	9	11.6	9	8.3	9	12.3	11

Intervention	SDS		5-HT		DA		NE		Adverse reaction	
	SUCRA	RANK	SUCRA	RANK	SUCRA	RANK	SUCRA	RANK	SUCRA	RANK
Tuina	16.8	9	70.5	3	90.3	1	55.8	5	68.7	2
rTMS	46.2	6	75.9	2	86.9	2	67.4	2	46.2	7
HBO	93.7	1	–	–	–	–	–	–	41.1	9
ACE	63.2	4	82.0	1	–	–	–	–	25.7	10
FB	-	-	51.3	6	39.7	5	–	–	65.9	3
MB	68.5	2	50.2	7	-	-	55.5	6	48.2	6
EA	58.1	5	61.8	4	79.9	3	69.9	1	64.2	4
MA	64.3	3	57.9	5	57.2	4	57.8	4	54.4	5
AS	45.6	7	35.1	8	24.2	6	61.9	3	42.6	8
CM	31.9	8	14.4	9	17.8	7	20.2	7	4.2	11
SI	11.8	10	1.0	10	4.0	8	11.5	8	88.7	1

### 3.5 Publication bias

Stata 16.0 was used to test for small-sample effects for each outcome indicator except for DA. Stata was also used to produce funnel plots. The funnel plots for AT, NE, SAS scores, and adverse events showed good symmetry, suggesting that the quality of the included studies was high and the possibility of publication bias was low. In terms of the total effective rate, there was a pronounced outlier on the left side. The exclusion of this outlier had no significant effect on the study results. Therefore, the probability of publication bias was considered low. The funnel plots for the remaining outcome indicators had poor symmetry, suggesting that there may have been some publication bias (Supplementary Figures S17–26).

### 3.6 Sensitivity analysis

To test the reliability and stability of this network meta-analysis, two sensitivity analyses were performed using Stata 16.0. First, four papers (39, 63, 67, 87) that were evaluated as high-risk in terms of literature quality were excluded, and sensitivity analyses were performed before and after exclusion. Second, considering that RCTs with small sample sizes may have affected the accuracy of the results, 14 studies (40, 54, 58, 61, 67, 72, 78, 80, 88, 92, 97, 107, 111, 117) with a sample size of less than 60 were excluded from the sensitivity analysis. Sensitivity analyses were only performed for some indicators (total effective rate, PSQI, SAS scores, and adverse reactions). The results show that there is little difference between the results before and after the exclusion of the two sensitivity analyses, indicating that the quality of the literature is good and that this network meta-analysis is reliable and stable.

## 4 Discussion

To the best of our knowledge, neither a systematic review nor a network meta-analysis of the efficacy and safety of external therapies in the treatment of insomnia has been reported. This is the first study to use a network meta-analysis to evaluate the effects of external therapies on sleep quality, psychological status, and neurotransmitters in patients with insomnia. In this network meta-analysis, we pooled evidence from 83 studies with 6,100 patients in total.

The total effective rate, PSQI as a subjective scale, and PSG indices (TST, SL, and AT) as objective indicators were used to assess the effectiveness of the external treatments in improving sleep quality. An in-depth analysis of the indicators revealed that the external therapies included in this study were superior to SI in terms of improving sleep quality, with Tuina therapy and rTMS consistently ranking highly. Tuina was the best intervention for improving the total effective rate, PSQI, and SL. The reason for the better effect of Tuina may be due to the activation of the corticotropin-releasing hormone (CRH)/CRH receptor type 1 (CRH/CRHR1) pathway and modulation of monoamine neurotransmitters (121, 122). rTMS was effective in increasing TST and reducing AT. Studies have shown that rTMS can reduce cortical excitability and increase melatonin secretion in the pineal gland, thereby restoring brain activity to a normal state (123). Although both Tuina and rTMS have good efficacy, after considering their safety, we found that rTMS was less safe, while Tuina was second only to SI in terms of safety. Therefore, the use of Tuina is a good choice when symptoms of sleep disorders are observed in patients.

Insomnia is also associated with many emotional and psychological changes, and prolonged poor-quality sleep not only affects quality of life, but also has many negative psychological effects on patients (124). Some studies have shown that the relationship between insomnia and depression and anxiety is bidirectional and that improvements in poor psychological states can also affect the effectiveness of insomnia treatment (125, 126). After analysis of relevant indicators, it was found that Tuina and rTMS, which were more effective in improving sleep disorder symptoms, were less effective in improving patients’ anxiety and depressive states. MB and HBO were the most effective in reducing SAS and SDS scores, second only to Tuina and rTMS in improving insomnia symptoms, and were found to be the most effective interventions for improving anxiety. Studies have shown that MB can promote blood circulation through warm stimulation, increase melatonin synthesis, improve mood, and promote sleep by regulating monoamine neurotransmitters, inhibitory neurotransmitters, and cytokines (127). HBO was effective in reducing depressive symptoms, which may be related to its ability to increase blood oxygen levels, improve blood supply to the brain, and restore neurological function (128, 129).

Monoamine neurotransmitters are important factors in the maintenance of the sleep–wake balance. The representative monoamine neurotransmitters 5-HT, DA, and NE are involved in the regulation of sleep activities and are closely related to the occurrence and development of insomnia (130, 131). We analyzed three outcome indicators, 5-HT, DA, and NE levels, and the results showed that Tuina and rTMS, which ranked high in the effectiveness index, also improved 5-HT, DA, and NE concentrations more effectively, with some positive correlations, which may underly their mechanisms of action. However, there is also a certain negative correlation; for example, EA is the most effective at reducing NE, but all of them are poorly ranked in terms of sleep quality and thus still need to be validated in a large number of clinical trials.

When we assessed the literature quality, we found that most studies used low-risk random assignment methods. The literature included in previous studies rarely mentioned blinding and allocation concealment or described them inaccurately. In this study, nearly half of the studies explicitly proposed the blind method and allocation concealment and described the specific implementation methods. The funnel plot showed some heterogeneity in some outcome indicators. Therefore, in order to identify the source of heterogeneity and improve the persuasiveness of the evidence, we conducted two sensitivity analyses of the total efficacy rate (as indicated by PSQI scores, SAS scores, and adverse reactions). After high-risk literature and small-sample studies were excluded, the overall results were still robust, indicating that the quality of the literature included in this study was acceptable.

However, this study had some limitations. First, owing to the relatively strict inclusion and exclusion criteria, RCTs with vagus nerve stimulation, transcranial direct current stimulation, and transcranial alternating current stimulation were not included; therefore, no statistical analysis of these therapies was conducted. Second, we only performed sensitivity analyses and not subgroup analyses because of limited evidence. Third, network diagrams may oversimplify complex relationships or overlook certain nuances in the NMA. Furthermore, Some objective indicators, such as slow wave sleep, REM sleep percentage, spindle and slow wave power and number, were not included in the analysis because external therapy reported less, which could not satisfy the statistical analysis. Finally, most included studies lacked follow-up data.

## 5 Conclusion

Altogether, most types of external treatments showed improvements in both subjective and objective sleep indices. External treatments are also highly effective in improving the psychological state and monoamine neurotransmitter levels in patients with insomnia. Tuina improves sleep disorder symptoms, and HBO and MB can be prioritized for insomnia treatment when patients have depression or anxiety as their main symptoms. The mechanism of action of external therapy for insomnia may be positively correlated with the regulation of monoamine neurotransmitters. However, this is not a one-to-one correlation. Limited by the quality of the included studies, the obtained conclusions must be verified.

## Data availability statement

The original contributions presented in this study are included in the article/Supplementary material. Further inquiries can be directed at the corresponding author.

## Author contributions

ZhenW: Methodology, Writing – original draft, Writing – review & editing. HX: Data curation, Software, Visualization, Writing – review & editing. ZhengW: Funding acquisition, Methodology, Software, Supervision, Writing – review & editing. HZ: Formal analysis, Investigation, Resources, Writing – review & editing. LZ: Conceptualization, Methodology, Supervision, Writing – review & editing. YW: Data curation, Formal analysis, Investigation, Writing – review & editing. ML: Conceptualization, Data curation, Validation, Writing – review & editing. YZ: Conceptualization, Funding acquisition, Visualization, Writing – original draft, Writing – review & editing.
