# Supplementary material for: Efficacy and safety of multiple external therapies in patients with insomnia: a systematic review and network meta-analysis
Source: Front Neurol. 2024 Jul 5;15:1297767. doi: 10.3389/fneur.2024.1297767 (PMC11258043; doi:10.3389/fneur.2024.1297767)
Supplement: Supplementary file 1 [file Data_Sheet_1.PDF]

Efficacy and safety of multiple external therapies in patients with insomnia: a systematic review and network meta-analysis

Supplementary materials

**The search strategies of each database**	**Figure 1; Table 1**	**Page 2-3**
**The characteristics of included 83 RCTs**	**Table 2**	**Page 4-6**
**Introduction to different Interventions**	**Table 3**	**Page 7**
**Risk of bias summary**	**Figure 2**	**Page 8**
**The results of heterogeneity test**	**Table 4**	**Page 9**
**Node-splitting test**	**Table 5-12**	**Page 10-13**
**Evidence network diagram**	**Figure 3-11**	**Page 14-15**
**The results of network meta-analysis**	**Figure 12-16**	**Page 16-17**
**Funnel plot**	**Figure 17-26**	**Page 18-19**
**Summary of results of adverse events**	**Table 12**	**Page 20**

Number	Search terms
1	exp Insomnia/Sleeplessness
2	exp Insomnia disorder/DIMS
3	Sleep initiation and maintenance disorders .ti,ab
4	Early awakening .ti,ab
5	or 1-4
7	Acupoints catgut embedding .ti,ab
8	Repetitive transcranial magnetic stimulation .ti,ab
9	Tuina .ti,ab
10	Hyperbaric oxygen .ti,ab
11	Electroacupuncture .ti,ab
12	Foot bath .ti,ab
13	Auricular stimulation .ti,ab
14	Moxibustion .ti,ab
15	Manual acupuncture .ti,ab
16	or 7-15
17	exp randomized controlled clinical trial .pt
18	exp controlled clinical trial .pt
19	randomised .ti,ab
20	randomized .ti,ab
21	randomly .ti,ab
22	trial .ti,ab
23	groups.ti,ab
24	or 17-23
25	exp animals/not humans.sh.
26	24 not 25
26	5 and 16 and 26

Figure 1 The Search Strategies of Pubmed

Table S1 Search Strategies of Network Meta-analysis

**Cochrane library**
#1 'Insomnia':ti,ab,kw OR 'Sleeplessness':ti,ab,kw OR 'Insomnia disorder':ti,ab,kw OR 'DIMS':ti,ab,kw OR 'Sleep initiation and maintenance disorders':ti,ab,kw OR 'Early awakening':ti,ab,kw
#2 Acupoints catgut embedding:ti,ab,kw OR Repetitive transcranial magnetic stimulation:ti,ab,kw OR Tuina:ti,ab,kw OR Hyperbaric oxygen:ti,ab,kw OR Electroacupuncture:ti,ab,kw OR Foot bath:ti,ab,kw OR Auricular stimulation:ti,ab,kw OR Moxibustion:ti,ab,kw OR Manual acupuncture:ti,ab,kw
#3 randomized controlled clinical trial:ti,ab,kw OR controlled clinical trial:ti,ab,kw OR randomised:ti,ab,kw OR groups:ti,ab,kw OR RCT:ti,ab,kw
#1 AND #2 AND #3

Web of science
#3 AND #2 AND #1<br>#3 TS=(Insomnia OR Sleeplessness OR Insomnia disorder OR DIMS OR Sleep initiation and maintenance disorders)<br>#2 TS=(Acupoints catgut embedding OR Repetitive transcranial magnetic stimulation OR Tuina OR Hyperbaric oxygen OR Electroacupuncture OR Foot bath OR Auricular stimulation OR Moxibustion OR Manual acupuncture)<br>#1 TS=(randomized controlled clinical trial OR controlled clinical trial OR randomised OR groups OR RCT)
**EMbase**
#3 AND #2 AND #1<br>#3 Insomnia:ab,ti OR Sleeplessness:ab,ti OR Insomnia disorder:ab,ti OR DIMS:ab,ti OR Sleep initiation and maintenance disorders:ab,ti OR<br>#2 Acupoints catgut embedding:ab,ti OR Repetitive transcranial magnetic stimulation:ab,ti OR Tuina:ab,ti OR Hyperbaric oxygen:ab,ti OR Electroacupuncture:ab,ti OR Foot bath:ab,ti OR Auricular stimulation:ab,ti OR Moxibustion:ab,ti OR Manual acupuncture:ab,ti<br>#1 'randomized controlled clinical trial':ab,ti OR 'controlled clinical trial':ab,ti OR 'randomised':ab,ti OR 'groups':ab,ti OR 'RCT':ab,ti
**China National Knowledge Infrastructure (CNKI)**
(SU=Shi mian OR SU=Bu mei OR SU=Shui mian zhang ai) AND (SU=Xue wei mai xian OR SU=Chong fu jing lu ci ci ji OR SU=Tui na OR SU=Gao ya yang OR SU=Dian zhen OR SU=Zu yu OR SU=Er xue ci ji OR SU=Ai jiu OR SU=Zhen jiu) AND (SU=Sui ji dui zhao shi yan OR SU=Sui ji OR SU=Fen zu)
**VIP Database**
M=(Shi mian OR Bu mei OR Shui mian zhang ai) AND M=(Xue wei mai xian OR Chong fu jing lu ci ci ji OR Tui na OR Gao ya yang OR Dian zhen OR Zu yu OR Er xue ci ji OR Ai jiu OR Zhen jiu) AND M=(Sui ji dui zhao shi yan OR Sui ji OR Fen zu)
**Chinese Biomedical Literature Database (CBM)**
(Shi mian[Zhai yao] OR Bu mei[Zhai yao] OR Shi mian zhang ai[Zhai yao]) AND (Xue wei mai xian[Zhai yao] OR Chong fu jing lu ci ci ji[Zhai yao] OR Tui na[Zhai yao] OR Gao ya yang[Zhai yao] OR Dian zhen[Zhai yao] OR Zu yu[Zhai yao] OR Er xue ci ji[Zhai yao] OR Ai jiu[Zhai yao] OR Zhen jiu[Zhai yao]) AND (Sui ji dui zhao shi yan[Zhai yao] OR Sui ji[Zhai yao] OR Fen zu[Zhai yao])
**Wanfang databases**
Zhu ti:(Shi mian or Bu mei or Shi mian zhang ai) and Zhu ti:(Xue wei mai xian or Chong fu jing lu ci ci ji or Tui na or Gao ya yang or Dian zhen or Zu yu or Er xue ci ji or Ai jiu or Zhen jiu) and Zhu ti:(Sui ji dui zhao shi yan or Sui ji or Fen zu)

Table 2 Basic characteristics of included studies

Included Studies	Mean age/years		Sample size（M/F）		Mean disease duration/year		Interventions		Duration	Outcome
	T	C	T	C	T	C	T	C	/month	Measures
Cao XL 2014	55.00±11.00	55.24±10.71<br>56.73±13.59	11/31	12/22<br>14/32	--	--<br>--	ACE	MA<br>CM	1month	①②⑨
Guo AS 2013	18～65	18～65	35	35	1～10	1～10	ACE	MA	6weeks	①②⑨
Li PZ 2021	54.92±8.35	52.30±7.00	9/17	12/15	66.54±29.03day	63.67±27.72day	ACE	HBO	2weeks	①②⑨
Xu F 2013	42.18±12.15	41.35±11.68<br>40.55±12.06	9/21	10/20<br>8/22	19.98±27.55day	20.65±26.97day<br>24.03±29.01day	ACE	MA<br>CM	1month	①②⑨
Li XR 2020	37±12	41±11	8/26	10/24	4.1±5.3	2.5±2.1	ACE	MA	6week	①②④⑤⑨
Chen YY 2020	51.20±9.27	50.43±10.00	10/20	11/19	--	--	ACE	MA	1month	①②⑥⑨
Yu WW 2017	67.25±6.71	68.00±7.56	14/17	16/17	2.76±1.56	2.64±1.49	rTMS	SI	10d	⑥⑦⑧⑨
Feng XJ 2017	44.1±8.3	45.1±7.8	12/31	14/23	8.8±1.4	7.6±1.9	rTMS	SI	10d	①②④⑤⑨
Yu XL 2022	62.62±5.65	62.83±5.72	29/12	31/10	0.96±0.43	0.95±0.42	rTMS	CM	1month	①③⑥⑨
Qi WY 2022	63.12±6.07	63.75±5.92	26/20	27/18	6.44±1.13month	6.37±1.09month	rTMS	CM	1month	②④⑤⑨
Mai DN 2016	43.56±21.01	41.57±22.95	16/14	13/17	5.34±0.16	7.43±0.11	rTMS	SI	2weeks	①②③
Ding L 2018	66.3±7.5	67.6±8.6	26/16	28/14	3.24±1.23	3.16±1.42	rTMS	SI	2weeks	②⑨
Kou XL 2019	46.2±0.55	43.7±0.61	24/34	25/32	11.3±2.56month	14.7±3.82month	rTMS	MA	10d	①②
Wang JX 2020	43.53±5.28	44.02±6.01	21/19	20/20	8.48±1.07month	8.51±1.11month	rTMS	CM	14d	①③⑥⑦⑧
Sheng W 2019	61.17±12.56	59.72±10.36	40/10	35/17	--	--	rTMS	EA	14d	③
Xu L 2021	48.34±3.04	44.23±3.54	9/21	10/20	20.11±10.11month	21.13±11.20month	rTMS	EA	1month	②③
Meng H 2017	18～59	18～59<br>18～59	15/14	16/14<br>15/16	--	--<br>--	rTMS	CM<br>SI	1month	①②⑨
Tang L 2014	42±13.01	44±9.8	14/21	14/20	13±6.2	16±5.7	rTMS	SI	14d	①②⑥⑦⑧
Ren YX 2022	72.15±2.89	72.08±2.96	20/29	25/24	12.13±1.55month	11.68±1.71month	rTMS	CM	1month	①②③④⑤⑥⑧⑨
Jiang CG 2013	48.31±8.45	48.11±7.51	21/24	19/26	11.23±4.32month	12.51±5.12	rTMS	CM	14d	②③
Huang ZY 2018	44.94±11.64	45.22±10.85	9/9	9/9	4.69±4.77	3.72±4.65	rTMS	SI	10d	②⑨
Wang J 2021	40.21±3.94	40.19±3.85	20/19	18/21	3.15±0.56	3.11±0.54	Tuina	CM	2month	②⑥⑦⑨
Yu LW 2017	71.6±11.4	69.1±9.7	9/49	11/47	2.32±1.98	2.46±2.01	Tuina	CM	4weeks	②③
Wei M 2020	39.31±11.41	37.52±11.05	11/18	8/19	3.67±2.95	3.43±2.73	Tuina	AS	4weeks	②③⑨
Tang HL 2015	44±10	46±10	14/24	12/26	21.0±17.6month	20.0±15.6month	Tuina	CM	4weeks	①②
Pan LK 2018	44.33±10.37	47.03±10.68	5/25	4/26	5.01±4.42	5.43±5.16	Tuina	CM	2weeks	①②④⑤
Zhou XB 2010	42.73±6.67	42.56±6.05	10/20	8/22	3.46±1.83	4.10±1.56	Tuina	MA	4weeks	①②⑨
Tan T 2014	39.6±8.0	37.3±10.0	18/12	20/10	--	————	Tuina	CM	20d	①②⑥⑧
Zheng HH 2022	59.1±6.1	58.7±5.3	15/16	17/14	3.9±1.2	3.4±1.1	HBO	CM	2month	②③
Pan Y 2016	59.25±11.52	60.15±10.23	11/9	12/8	2.96±2.84	3.06±2.12	HBO	CM	2month	②③
Liao HY 2010	--	--	36	35	--	————	HBO	CM	1month	①②⑨
Kuang AH 2009	26～68	26～68	42	42	2～72month	2～72month	HBO	CM	20d	②
Zhu Y 2020	53.6±6.7	52.8±7.1	14/11	15/10	--	————	HBO	CM	3month	④⑤
Sun YZ 2021	58.63±8.58	58.33±8.45	12/18	14/16	6.67±2.75	6.63±3.20	EA	MA	4weeks	①②⑥⑧⑨
Wang XQ 2021	69±4	69±5	13/17	11/18	19.9±6.0	19.7±5.7	EA	SI	4weeks	②⑦⑨
Yeung WF 2011	47.5±8.5	46.7±9.7<br>50.1±9.1	6/20	7/19<br>3/23	--	--<br>--	EA	MA<br>SI	3weeks	②③⑤⑨

Yeung WF 2009	48.3±9.5	47.8±8.6	8/22	6/24	--	--	EA	SI	3weeks	②③⑨
Li SS 2020	52.12±4.19	53.07±3.81	42	42	39.19±15.00	40.21±16.00	EA	SI	2month	②③④⑤⑨
Lee B 2020	51.78±4.92	52.00±4.95	9/40	9/43	7.63±5.35	7.24±5.36	EA	SI	3month	②③⑨
Yin X 2020	47.30±14.89	49.80±15.13<br>46.77±15.57	11/19	10/20<br>11/19	5.67±5.70	7.48±6.23<br>5.89±5.64	EA	MA<br>SI	3month	②③⑤⑨
Wu WZ2021	41±10	42±10	12/18	10/19	13.4±5.3month	14.2±4.8month	EA	CM	4weeks	①②
Liu H 2022	49.2±2.3	49.4±2.3	30	30	12.6±6.1month	12.5±6.0month	MB	MA	1month	①②⑨
Lu XX 2020	57.22±2.43	58.23±2.23	3/11	2/12	6.28±1.62	6.54±1.71	MB	CM	1month	①②⑨
Li Q 2020	36.11±7.8	35.59±7.56	23/23	25/21	12.84±2.34	12.56±2.12	MB	CM	28d	①②③⑥
Li LC 2018	34.25±12.06	34.11±12.01	12/18	11/19	7.23±1.11	7.36±1.25	MB	CM	15d	②④⑤
Wu SX 2019	43.82±9.35	43.25±8.34	23/17	23/17	10.30±7.27month	10.35±7.09	MB	CM	1month	①②④⑨
Wang J 2021	60.52±8.14	61.00±8.76	22/18	25/15	43.32±8.97d	43.58±8.76d	MB	CM	28d	①②⑥⑨
Li J 2020	49.04±8.74	49.02±8.52	12/38	10/40	5.00±4.00	5.84±2.96	MB	Tuina	1month	①②⑨
Li LC 2018	34.25±12.06	34.11±12.01	18/12	19/11	7.23±1.11month	7.36±1.25month	MB	CM	15d	①②
Li D 2021	64.37+6.52	63.93±6.05	26/14	25/15	3.42+0.85month	3.41±0.82month	MB	MA	30d	①④⑤
Chen Y 2021	64.14±14.39	65.93±11.52	14/14	13/15	4.64±1.40month	4.71±1.51month	MB	CM	30d	①②⑥⑧
Chen Q 2013	42.17±10.03	41.43±9.89	23/45	20/47	12.00±8.87month	10.34±7.76month	MB	CM	1month	②⑤⑨
Li SH 2015	20～65	20～65	30	30	--	--	FB	CM	1month	①②
Cao DF 2018	20～60	20～60	15/20	13/22	--	--	FB	CM	1month	②
Wang XB 2021	71.58±4.16	71.61±4.12	9/7	8/8	4.58±1.47	4.55±1.45	FB	CM	4weeks	①⑥⑦⑨
Zhang H 2019	46.3±10.7	46.2±10.6	29/24	26/20	--	--	FB	SI	4weeks	②④
An XM 2016	--	--	30	30	--	--	FB	AS	4weeks	①②⑥⑦
Li M 2018	28～68	28～68<br>28～68	20	20<br>20	--	--<br>--	FB	Tuina<br>CM	4weeks	①
Xie C 2018	48±7	46±8	25/20	23/22	16.89±3.87month	16.98±4.02month	MA	CM	30d	①④⑥
Xi HQ 2021	44±12	41±12	12/17	13/16	6.6±4.4	5.7±4.7	MA	SI	4weeks	②⑨
PAN Y 2017	55.5±3.1	55.7±3.2	23/17	24/16	1±0.4	1±0.2			2weeks	②⑦⑧
Zhang LX 2020	18～65	18～65	30	30	--	--	MA	SI	10d	①②⑤⑨
Liang FJ 2020	56.07±10.46	60.10±11.22	8/22	15/15	--	--	MA	CM	28d	②⑥⑨
Liu JX 2017	62.08±7.8	63.02±7.7	21/14	20/15	2.59±0.24month	2.74±0.2month	MA	CM	4weeks	①②③④⑤⑨
Guo Q 2021	49.83±3.65	50.20±4.10	30	30	36.53±13.61month	33.93±14.79month	MA	CM	8weeks	①②⑥⑧
Yu L 2022	49.79±14.76	48.41±16.98	24/54	21/54	--	--	MA	CM	4weeks	①②④⑤⑨
Wang C 2021	18～75	18～75	13/28	10/31	0.3～40	0.3～40	MA	SI	4weeks	②③⑨
Yeung WF 2021	42.2±12.8	42.0±13.3	56	56	5.0±5.8	5.5±6.4	MA	SI	4weeks	③⑨
Fu C 2017	52.0±5.3	52.5±5.9	37	37	--	--	MA	SI	3weeks	②③
Liu CY 2021	47.17±14.08	45.59±12.65	13/16	10/17	2.71±3.22month	2.90±3.55month	MA	SI	4weeks	②⑥⑨
Zhang LX 2020（2）	36.6±14.4	39.2±13.8	26/22	27/21	20.6±18.9month	21.6±16.9month	MA	SI	2weeks	②④⑤⑨
Yin X 2017	39.7±12.9	37.3±15.1	17/19	15/21	--	--	MA	SI	4weeks	③④⑤⑨
Zhong SQ 2022	52.91±7.21	52.56±6.95	18/12	17/13	--	--	AS	SI	14d	①②③
Chen XY 2021	58.64±6.43	58.21±7.03	14/12	13/13	--	--	AS	SI	14d	⑥⑧
Jiang YB 2018	42.13±3.91	36.69±3.89<br>38.73±4.01	7/28	7/23<br>8/22	--	--<br>--	AS	CM<br>SI	7d	②③
Chen H 2022	20.0±10.0	21.0±3.0	8/28	5/32	22.0±6.0month	16.0±6.0month	AS	Tuina	8weeks	①②⑨

Wu YC 2022	52.34±13.07	55.90±13.25	25/39	38/31	--	--	AS	SI	8weeks	②⑨
Xiang Y 2022	60.20±7.61	63.57±8.91	9/21	13/17	--	--	AS	CM	4weeks	①②④⑤⑨
Zang LL 2014	48.7±12.8	49.2±12.5	39	40	--	--	AS	CM	40d	①②④⑤
Luo MF 2015	23～67	23～67	25	25	--	--	AS	SI	4weeks	②⑥⑦
Zhang J 2022	28.89±4.87	27.03±3.41	28	28	6.32±1.51month	6.46±1.68month	AS	CM	7d	②③
Cao J 2020	35.63±3.32	35.78±3.05	16/19	14/21	16.5±3.9month	16.1±4.5month	AS	MA	4weeks	①②
Guo X 2019	57.13±8.21	58.37±7.87<br>58.54±8.29	18/12	17/13<br>14/12	--	--<br>--	AS	MB<br>CM	14d	①②

Note: T. Treatment group ; C. Control group; --. It was not mentioned; ACE.acupoints catgut embedding; rTMS.Repetitive transcranial magnetic stimulation; HBO.Hyperbaric oxygen; EA.Electroacupuncture; MB.Moxibustion; FB.Foot bath; MA.Manual acupuncture; AS.Auricular stimulation; CM.conventional medicine; SI.Sham intervention; ①TER.Total effective rate; ②PSQI.Pittsburgh sleep quality index; ③PSG.Polysomnography; ④SAS.Self-rating anxiety scale; ⑤SDS.Self-rating depression scale; ⑥5-HT.5-hydroxytryptamine; ⑦DA.Dopamine; ⑧NE.Norepinephrine; ⑨Adverse reaction.

Table 3 Introduction to different Interventions

Interventions	Abbreviation	Description
Acupoints catgut embedding	ACE	Under the guidance of acupuncture meridians theory, it is a method to bury medical catgut or other absorbable thread body into the corresponding acupoint area through needles, and stimulate the acupoints persistently and gently through various factors to dredge the meridians qi and blood to treat diseases.
repetitive transcranial magnetic stimulation	rTMS	A treatment technique in which a pulsed magnetic field is repeatedly applied to the local central nervous system outside the skull to change the membrane potential of cortical nerve cells and generate an induced current, which affects the metabolism and electrical activity of nerves in the brain, thereby causing a series of physiological and biochemical reactions.
Tuina	Tuina	The doctor uses his hands to act on the patient's body surface, the injured part, the discomfort place, the specific acupoint, the painful place, the specific use of pushing, holding, pressing, rubbing, kneading, pinching, point, patting and other forms of various techniques and forces, in order to achieve the purpose of treating diseases.
Hyperbaric oxygen	HBO	A method of treating disease by using a hyperbaric chamber where the patient is placed under high pressure (above normal pressure) and breathing pure or highly concentrated oxygen.
Electroacupuncture	EA	EA is a method of preventing and treating disease by combining needle and electrical stimulation by passing a trace current close to the body's bioelectricity through the needle tool after the needle has been inserted into the acupoint to obtain Qi.
Moxibustion	MB	MB is a treatment method that uses moxa leaves to make moxa sticks, moxa pillars, which produce moxa heat to stimulate acupuncture points or specific areas of the body for the purpose of preventing and treating disease.
Hyperbaric oxygen	HBO	A method of treating disease by using a hyperbaric chamber where the patient is placed under high pressure (above normal pressure) and breathing pure or highly concentrated oxygen.
Foot bath	FB	Foot bath is a kind of health care therapy, the use of 40-50 degrees of hot water to soak the foot (sometimes can add drugs), can promote human blood circulation, to improve the human meridians, promote human health purposes.
Manual acupuncture	MA	A needle is inserted into a specific acupoint at a certain angle according to the theory of traditional Chinese medicine. Acupuncture techniques such as twisting and lifting are used to stimulate specific parts of the body to treat diseases.
Auricular stimulation	AS	Auricular point stimulation refers to the use of short acupuncture or other methods to stimulate specific areas of the ear (namely auricular points) to prevent and treat diseases.
Conventional medicine	CM	A needle is inserted into a specific acupoint at a certain angle according to the theory of traditional Chinese medicine. Acupuncture techniques such as twisting and lifting are used to stimulate specific parts of the body to treat diseases.
Conventional medicine	CM	All drugs must be approved by the US Food and Drug Administration or the State Drug Administration of China and conform to the guidelines or expert consensus recommendations for the treatment of insomnia. To reduce heterogeneity, only benzodiazepines are included in conventional medicine.
Sham intervention	SI	It is a sham intervention that has no therapeutic effect and has a substitution and comforting effect.

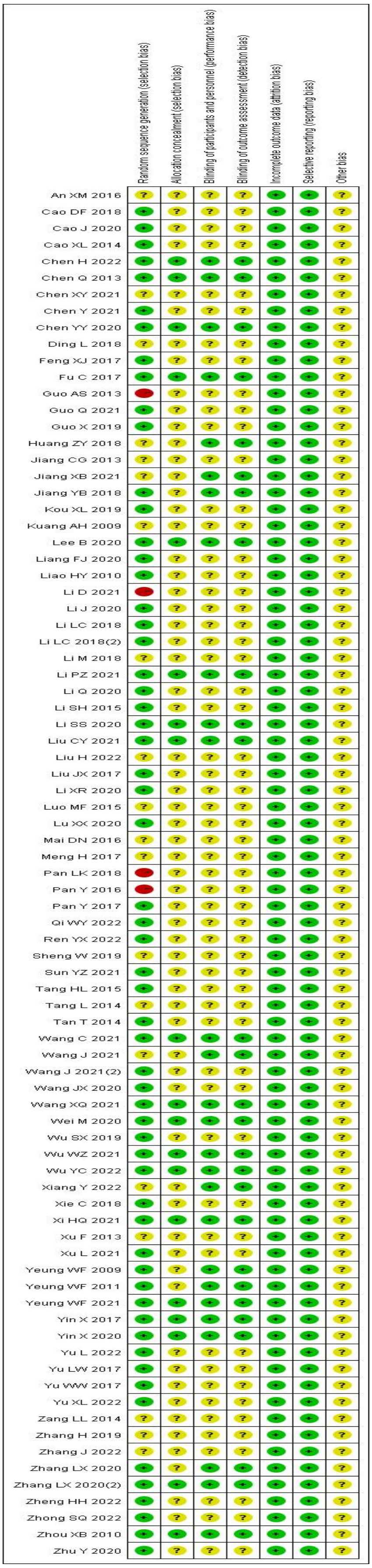

Figure 2 Risk of bias summary

Table 4 The results of heterogeneity test

Outcome indicator	Degrees of freedom	P	I^2	Tau-squared
Total effective rate	49	0.000	65.8%	0.894
PSQI	75	0.000	92.6%	0.852
TST	25	0.000	78.7%	0.214
SL	21	0.000	93.3%	0.838
AT	15	0.000	85.0%	0.354
SAS	17	0.000	92.8%	0.753
SDS	20	0.000	93.6%	0.914
5-HT	19	0.000	97.7%	4.412
DA	8	0.000	98.7%	17.62
NE	9	0.000	96.7%	2.660
Adverse reaction	42	0.000	74.8%	2.210

Node-splitting test

Table 5 Node-splitting test of total effective rate

Side	Direct		Indirect		Difference		P
	Coef.	Std. Err.	Coef.	Std. Err.	Coef.	Std. Err.	
CM VS SI	-3.3016	1.0149	-2.5579	0.6338	-0.7437	1.1979	0.535
CM VS ACE	1.0586	0.7044	1.7362	0.6697	-0.6775	0.9513	0.476
CM VS rTMS	1.5098	0.5146	1.9609	0.7530	-0.4510	0.9168	0.623
CM VS Tuina	2.3944	0.6443	1.1653	0.6227	1.2291	0.8813	0.163
CM VS HBO	1.6526	0.9463	1.2579	1.0826	0.3947	1.4379	0.784
CM VS EA	-0.4054	1.1800	2.1164	1.0665	-2.5219	1.5906	0.113
CM VS MB	1.4104	0.3758	0.4003	0.6357	1.0101	0.7394	0.172
CM VS FB	1.7736	0.6253	0.4329	0.9517	1.3407	1.1355	0.238
CM VS MA	1.0880	0.4217	0.9321	0.4708	0.1559	0.6332	0.806
CM VS AS	0.2845	0.5186	1.2801	0.5710	-0.9955	0.7735	0.198
SI VS rTMS	4.3847	0.6129	4.5123	0.9912	-0.1275	1.1862	0.914
SI VS MA	3.7658	1.0763	3.7925	0.6578	-0.0267	1.2614	0.983
SI VS AS	3.1372	1.6512	3.5674	0.6803	-0.4301	1.7859	0.810
ACE VS HBO	-0.1053	0.9364	0.2890	1.0912	-0.3943	1.4380	0.784
ACE VS MA	-0.4534	0.5315	-0.1855	1.0416	-0.2678	1.1501	0.816
rTMS VS MA	-1.1394	0.8718	-0.4380	0.5468	-0.7014	1.0291	0.496
Tuina VS MB	0.1129	0.8690	-1.0124	0.6294	1.1253	1.0730	0.294
Tuina VS FB	-1.1132	1.3405	-0.1618	0.7788	-0.9514	1.5631	0.543
Tuina VS MA	-3.1374	1.6381	-0.4790	0.5521	-2.6584	1.7287	0.124
Tuina VS AS	0.1823	0.9659	-1.5530	0.6370	1.7353	1.1571	0.134
EA VS MA	-1.0076	1.0206	1.5144	1.2199	-2.5220	1.5905	0.113
CM VS SI	-3.3016	1.0149	-2.5579	0.6338	-0.7437	1.1979	0.535
MB VS AS	-0.1433	0.9182	-0.5083	0.5534	0.3649	1.0723	0.734
FB VS AS	-0.1643	0.9296	-0.9320	0.7382	0.7676	1.1871	0.518
MA VS AS	-0.2559	1.0320	-0.2850	0.5163	0.0291	1.1540	0.980

Table 6 Node-splitting test of PSQI

Side	Direct		Indirect		Difference		P
	Coef.	Std. Err.	Coef.	Std. Err.	Coef.	Std. Err.	
CM VS SI	3.8434	1.3831	1.5894	0.6226	2.2540	1.5164	0.137
CM VS ACE	-3.3163	1.4059	-2.1196	1.0803	-1.1966	1.7753	0.500
CM VS rTMS	-2.7277	0.9607	-3.2763	0.9578	0.5486	1.3566	0.686
CM VS Tuina	-3.8253	0.9882	-2.4999	1.0791	-1.3253	1.4633	0.365
CM VS HBO	-2.2175	0.9907	-2.8427	2.2442	0.6251	2.4532	0.799
CM VS EA	-2.7100	1.9524	-1.1641	0.8538	-1.5458	2.1309	0.468
CM VS MB	-1.7394	0.6494	-3.7124	1.3068	1.9730	1.4594	0.176
CM VS FB	-3.5765	1.4108	-0.7479	1.5000	-2.8286	2.0592	0.170
CM VS MA	-2.1249	0.7305	-1.3701	0.7203	-0.7547	1.0259	0.462
CM VS AS	-0.0311	0.8762	-0.9078	0.8828	0.8766	1.2444	0.481
SI VS rTMS	-5.0199	0.8113	-4.8937	1.0133	-0.1262	1.2983	0.923
SI VS FB	-2.4899	1.9431	-4.9854	1.2957	2.4954	2.3356	0.285
SI VS MA	-4.4779	0.6953	-2.6830	0.8073	-1.7948	1.0653	0.092
SI VS AS	-1.8331	1.0003	-2.9644	0.9392	1.1312	1.3724	0.410
ACE VS HBO	-0.1899	2.0442	0.4361	1.3565	-0.6261	2.4534	0.799
ACE VS MA	0.7670	0.8933	1.06033	1.8590	-0.2932	2.0661	0.887
rTMS VS EA	1.5300	1.9176	1.6055	0.9358	-0.0755	2.1337	0.972
rTMS VS MA	1.9500	1.9452	1.1562	0.7581	0.7937	2.0877	0.704
Tuina VS MB	-0.5699	1.8884	1.5369	0.9775	-2.1069	2.1264	0.322
Tuina VS MA	3.1799	2.0420	1.1432	0.9061	2.0367	2.2340	0.362
Tuina VS AS	1.6206	1.4209	3.3539	1.0333	-1.7332	1.7568	0.324
MB VS MA	2.7399	1.8458	-0.0457	0.7914	2.7857	2.0083	0.165
MB VS AS	0.6484	1.9047	1.8845	0.8919	-1.2361	2.1033	0.557
FB VS AS	0.8999	2.0970	2.1231	1.3012	-1.2231	2.4679	0.62
MA VS AS	-0.5800	2.2809	1.4719	0.7408	-2.0519	2.3982	0.392

Table 7 Node-splitting test of TST

Side	Direct		Indirect		Difference		P
	Coef.	Std. Err.	Coef.	Std. Err.	Coef.	Std. Err.	
CM VS SI	-37.3742	37.3957	-3.1976	20.7629	-34.1766	42.7500	0.424
CM VS rTMS	40.4104	22.6679	26.5856	32.9386	13.8248	39.9841	0.730
CM VS Tuina	20.2000	31.7305	46.0702	38.4322	-25.8701	49.8383	0.604
CM VS EA	69.5500	30.0596	4.1336	23.0988	65.4162	37.9096	0.084
CM VS AS	2.01399	23.6282	44.9757	28.9316	-42.9617	37.3763	0.250
SI VS rTMS	43.2800	35.7769	48.9876	23.6107	-5.7076	42.8653	0.894
SI VS EA	19.7104	14.7151	67.7170	31.4037	-48.0065	34.7627	0.167
SI VS MA	36.3859	13.6752	58.0762	32.8095	-21.6903	35.6142	0.543
SI VS AS	51.9594	22.2420	-17.5855	33.1901	69.5449	39.9875	0.082
rTMS VS EA	-13.1700	31.8279	-22.8828	27.5989	9.7123	42.1274	0.818
Tuina VS AS	-22.4799	32.3682	3.4164	37.9040	-25.8963	49.8439	0.603
EA VS MA	-23.7413	23.7349	36.7093	20.3935	-60.4506	31.2674	0.053

Table 8 Node-splitting test of SL

Side	Direct		Indirect		Difference		P
	Coef.	Std. Err.	Coef.	Std. Err.	Coef.	Std. Err.	
CM VS rTMS	-9.2490	4.6391	-1.1883	10.2646	-8.0606	11.2644	0.474
CM VS Tuina	-3.6300	8.0756	-28.1707	11.4959	24.5407	14.0489	0.081
CM VS AS	5.3400	9.1115	-12.2095	8.0137	17.5495	12.1342	0.148
SI VS rTMS	-8.5600	9.5967	-14.4422	6.7239	5.8822	11.7178	0.616
SI VS EA	-2.9866	6.7893	1.0157	9.3350	-4.0024	11.4771	0.727
rTMS VS EA	9.9163	7.0696	12.6076	9.3039	-2.6912	11.6837	0.818
Tuina VS AS	18.0399	9.3784	-6.5065	10.4612	24.5465	14.0497	0.081
EA VS MA	-24.1160	15.4801	-6.9430	6.8768	-17.1729	16.8421	0.308

Table 9 Node-splitting test of AT

Side	Direct		Indirect		Difference		P
	Coef.	Std. Err.	Coef.	Std. Err.	Coef.	Std. Err.	
CM VS SI	1.3362	1.2964	0.2768	1.0547	1.0593	1.6643	0.524
CM VS rTMS	-1.4995	0.6546	-0.4865	1.3655	-1.0130	1.5141	0.503
CM VS Tuina	-0.7600	0.8835	-2.2931	1.4253	1.5331	1.6770	0.361
CM VS MA	-0.6200	0.9060	0.2206	1.7744	-0.8406	1.9923	0.673
CM VS AS	-1.0677	1.2132	-0.5030	1.2435	-0.5646	1.7455	0.746
SI VS rTMS	-1.1199	2.5621	-2.1046	0.8392	0.9846	2.6961	0.715
SI VS EA	-1.0465	0.9087	-2.1690	1.3686	1.1224	1.6398	0.494
SI VS MA	-0.1757	1.9423	-1.5442	1.2342	1.3685	2.3234	0.556
SI VS AS	-2.4101	1.2063	0.1917	1.5944	-2.6019	2.0060	0.195
rTMS VS EA	0.4877	0.6576	1.1677	1.3543	-0.6800	1.5054	0.651
Tuina VS AS	0.8699	0.9166	-0.6660	1.4054	1.53606	1.6779	0.360
EA VS MA	0.49651	1.92559	0.14966	1.1862	0.34680	2.28496	0.879

Table 10 Node-splitting test of SAS

Side	Direct		Indirect		Difference		P
	Coef.	Std. Err.	Coef.	Std. Err.	Coef.	Std. Err.	
CM VS rTMS	-5.6521	3.6678	6.7283	6.9590	-12.3805	7.8660	0.116
CM VS MB	-6.4449	3.5702	-18.7048	5.5787	12.2596	6.6234	0.064
CM VS MA	-6.0003	3.4816	-4.2956	5.5745	-1.7046	6.5725	0.795
SI VS rTMS	-4.1600	5.2133	-16.5516	5.8946	12.3916	7.8693	0.115
SI VS EA	-3.1199	5.6707	-13.2674	313.004	10.1474	313.057	0.974
SI VS FB	-2.1300	5.6043	-13.1260	212.545	10.9960	212.619	0.959
SI VS MA	-14.9707	3.7388	-2.5701	6.9093	-12.4005	7.8556	0.114
ACE VS MA	-1.2899	5.7033	-11.0491	308.207	9.7591	308.261	0.975
MB VS MA	11.2299	4.9119	-1.0473	4.4461	12.2773	6.6253	0.064

Table 11 Node-splitting test of SDS

Side	Direct		Indirect		Difference		P
	Coef.	Std. Err.	Coef.	Std. Err.	Coef.	Std. Err.	
CM VS rTMS	-5.2458	5.0459	7.3222	8.9116	-12.5680	10.2410	0.220
CM VS MB	-2.7421	5.0088	-16.7151	8.1302	13.9730	9.5493	0.143
CM VS MA	-6.1227	5.3672	-4.3724	6.7486	-1.7503	8.6227	0.839
SI VS rTMS	-0.9500	7.1071	-13.5417	7.3856	12.5917	10.2498	0.219
ACE VS MA	0.82999	7.2725	-10.9075	242.056	11.7375	242.164	0.961
MB VS MA	8.4599	6.9088	-5.5539	6.6031	14.0139	9.5568	0.143

Table 12 Node-splitting test of adverse reaction

Side	Direct		Indirect		Difference		P
	Coef.	Std. Err.	Coef.	Std. Err.	Coef.	Std. Err.	
CM VS ACE	-0.4913	1.0817	-1.0844	0.9452	0.5930	1.4261	0.678
CM VS rTMS	-1.4744	0.6579	-1.3802	0.9950	-0.0941	1.1939	0.937
CM VS Tuina	-0.7487	1.3419	-3.0461	1.1501	2.2973	1.7673	0.194
CM VS HBO	-1.0443	1.2043	-1.6813	1.8047	0.6370	2.1696	0.769
CM VS MB	-1.5236	0.6702	-1.2117	1.6009	-0.3119	1.7355	0.857
CM VS MA	-1.8545	0.6118	-1.3421	0.7605	-0.5123	0.9849	0.603
CM VS AS	-2.7009	2.2613	-1.5642	1.0192	1.5642	2.4804	0.528
SI VS Tuina	0.9765	0.6482	1.4704	1.0108	-0.4938	1.2038	0.682
SI VS EA	0.6573	0.5894	0.6618	1.2287	-0.0044	1.3682	0.997
SI VS MA	0.9151	0.5063	0.9142	0.7869	0.0008	0.9379	0.999
SI VS AS	0.08855	1.0920	3.1483	1.4117	-3.0597	1.7848	0.086
ACE VS HBO	-0.7732	1.6327	-0.1360	1.4289	-0.6371	2.1697	0.769
ACE VS MA	-0.6069	0.7644	-1.7193	1.5998	1.1123	1.7992	0.536
Tuina VS MB	1.1186	1.9437	0.4113	1.1679	0.7072	2.2676	0.755
Tuina VS MA	-4.2909	2.2653	0.5102	1.0191	-0.5102	2.4839	0.837
Tuina VS AS	1.9153	1.3011	-0.6986	1.4606	2.6140	1.9561	0.181
EA VS MA	0.3431	0.7701	0.1455	0.8722	0.1976	1.1626	0.865
MB VS MA	-3.3509	2.2652	-0.1958	0.7919	0.1958	2.3997	0.935

Evidence network diagram

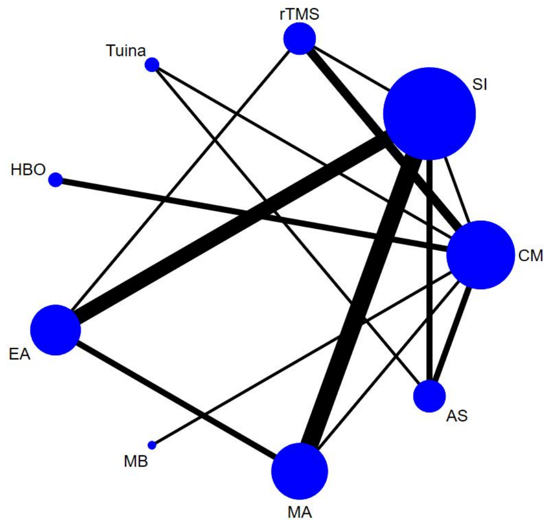

Figure 3 Network diagram of TST

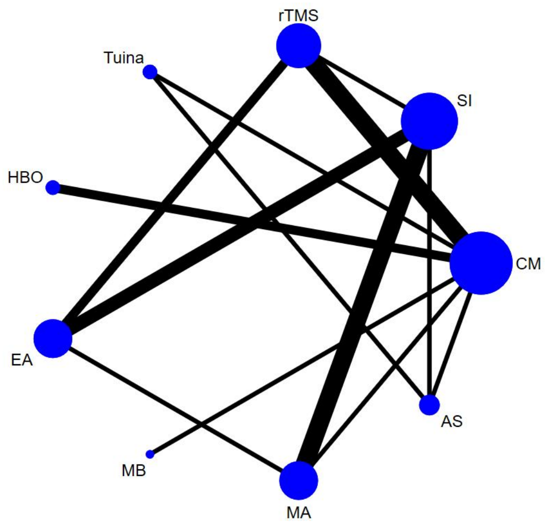

Figure 4 Network diagram of SL

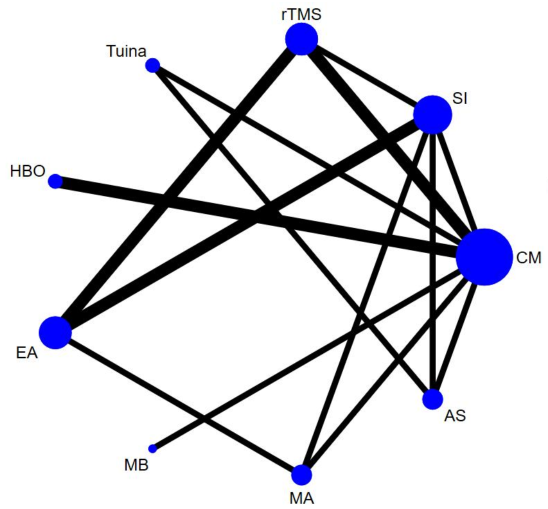

Figure 5 Network diagram of AT

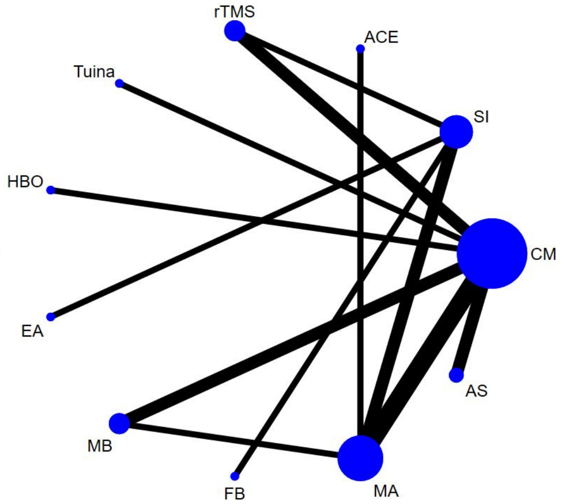

Figure 6 Network diagram of SAS

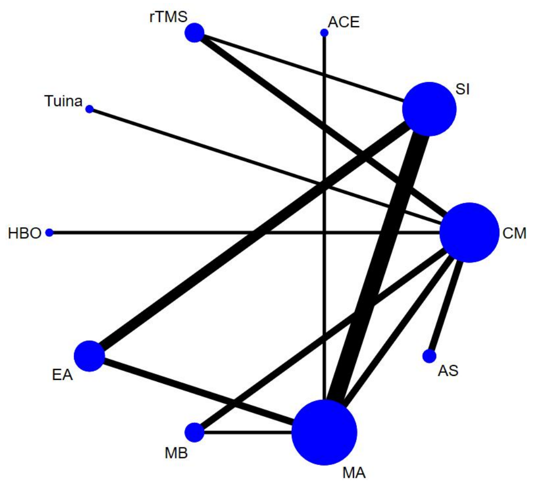

Figure 7 Network diagram of SDS

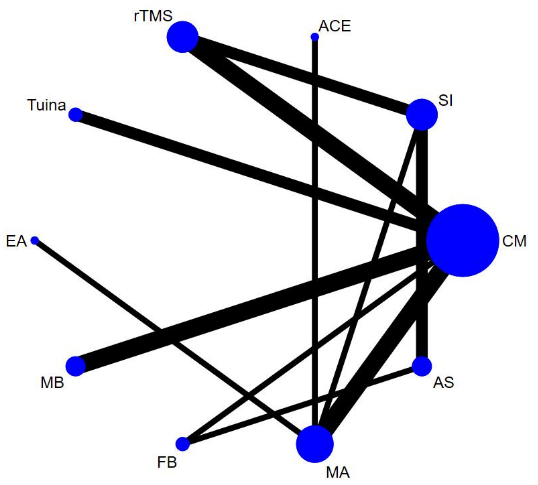

Figure 8 Network diagram of 5-HT

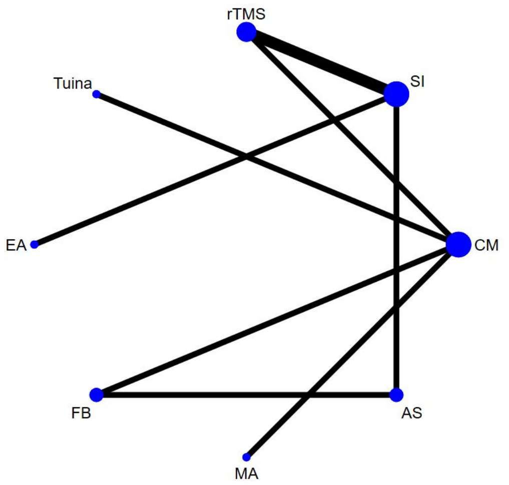

Figure 9 Network diagram of DA

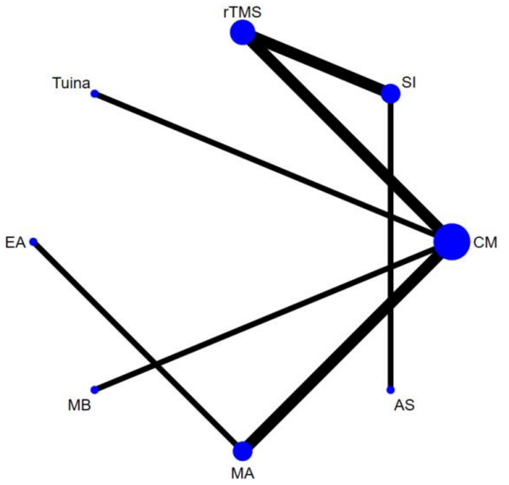

Figure 10 Network diagram of NE

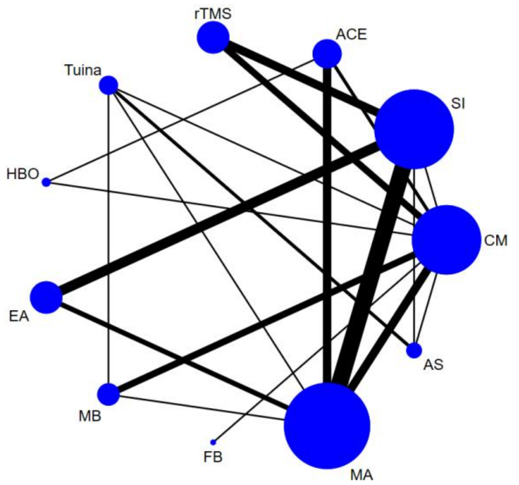

Figure 11 Network diagram of adverse reaction

The results of network meta-analysis

	TST										
PSQI	Tuina	3.02 (-50.71, 56.74)	----	26.65 (-48.92, 102.22)	----	-0.05 (-76.12, 76.02)	11.78 (-41.39, 64.95)	19.66 (-34.99, 74.30)	13.20 (-31.10, 57.49)	29.05 (-14.93, 73.02)	48.21 (-2.60, 99.02)
	-0.22 (-2.10, 1.66)	rTMS	----	29.67 (-40.63, 99.96)	----	2.96 (-67.87, 73.80)	14.79 (-24.58, 54.16)	22.67 (-14.82, 60.16)	16.21 (-27.31, 59.74)	32.06 (-2.06, 66.19)	51.23 (15.57, 86.89)
	-0.66 (-2.79, 1.48)	-0.44 (-2.43, 1.55)	ACE	----	----	----	----	----	----	----	----
	-0.90 (-3.16, 1.36)	-0.68 (-2.86, 1.50)	-0.25 (-2.45, 1.96)	HBO	----	26.70 (-60.65, 114.05)	14.87 (-56.30, 86.04)	6.99 (-65.40, 79.39)	13.45 (-56.84, 83.75)	2.40 (-59.06, 63.86)	21.56 (-48.40, 91.53)
	-0.97 (-3.39, 1.45)	-0.75 (-3.06, 1.55)	-0.32 (-2.88, 2.25)	-0.07 (-2.75, 2.61)	FB	----	----	----	----	----	----
	-1.09 (-2.79, 0.61)	-0.87 (-2.58, 0.84)	-0.44 (-2.42, 1.55)	-0.19 (-2.29, 1.91)	-0.12 (-2.42, 2.18)	MB	11.83 (-59.87, 83.53)	19.71 (-53.21, 92.63)	13.25 (-57.58, 84.08)	29.10 (-32.97, 91.17)	48.26 (-22.24, 118.77)
	-1.48 (-3.10, 0.14)	-1.26 (-2.64, 0.12)	-0.82 (-2.39, 0.74)	-0.58 (-2.54, 1.39)	-0.51 (-2.67, 1.65)	-0.39 (-1.82, 1.05)	MA	7.88 (-22.45, 38.21)	1.42 (-39.12, 41.96)	17.27 (-18.62, 53.16)	36.43 (13.49, 59.38)
	-1.81 (-3.82, 0.20)	-1.59 (-3.23, 0.05)	-1.15 (-3.23, 0.93)	-0.91 (-3.21, 1.39)	-0.84 (-3.25, 1.57)	-0.72 (-2.58, 1.15)	-0.33 (-1.79, 1.13)	EA	6.46 (-35.80, 48.72)	9.39 (-28.87, 47.65)	28.55 (3.57, 53.54)
	-2.75 (-4.39, -1.12)	-2.54 (-4.16, -0.91)	-2.10 (-4.07, -0.13)	-1.85 (-3.98, 0.27)	-1.78 (-3.94, 0.37)	-1.66 (-3.24, -0.09)	-1.28 (-2.66, 0.10)	-0.95 (-2.71, 0.81)	AS	15.85 (-18.28, 49.98)	35.02 (-1.30, 71.33)
	-3.22 (-4.65, -1.79)	-3.00 (-4.32, -1.68)	-2.56 (-4.24, -0.89)	-2.32 (-4.08, -0.55)	-2.25 (-4.28, -0.22)	-2.13 (-3.28, -0.98)	-1.74 (-2.74, -0.74)	-1.41 (-2.94, 0.11)	-0.47 (-1.68, 0.75)	CM	19.16 (-14.27, 52.60)
	-5.19 (-6.89, -3.48)	-4.97 (-6.20, -3.74)	-4.53 (-6.34, -2.73)	-4.29 (-6.34, -2.23)	-4.22 (-6.33, -2.10)	-4.10 (-5.64, -2.55)	-3.71 (-4.76, -2.66)	-3.38 (-4.72, -2.04)	-2.43 (-3.77, -1.10)	-1.97 (-3.09, -0.85)	SI

Figure 12 Network meta-analysis of PSQI and TST［MD(95%CI)］

	AT								
SL	Tuina	0.13 (-1.66, 1.92)	-0.02 (-3.43, 3.38)	-0.06 (-2.33, 2.20)	-0.73 (-2.82, 1.36)	-0.41 (-1.90, 1.07)	-1.18 (-2.64, 0.27)	-0.49 (-2.44, 1.47)	-1.88 (-3.85, 0.09)
	-3.97 (-19.83, 11.88)	rTMS	-0.15 (-3.43, 3.12)	-0.19 (-2.26, 1.87)	-0.86 (-2.64, 0.93)	-0.54 (-2.42, 1.33)	-1.31 (-2.42, -0.20)	-0.62 (-1.72, 0.49)	-2.01 (-3.56, -0.46)
	-3.63 (-24.84, 17.57)	0.34 (-17.40, 18.08)	HBO	0.04 (-3.50, 3.58)	-0.70 (-4.14, 2.73)	-0.39 (-3.87, 3.09)	-1.16 (-4.24, 1.92)	-0.46 (-3.85, 2.92)	-1.86 (-5.31, 1.59)
	-4.00 (-26.56, 18.56)	-0.02 (-19.37, 19.33)	-0.36 (-23.99, 23.27)	MB	-0.66 (-2.98, 1.65)	-0.35 (-2.73, 2.02)	-1.12 (-2.86, 0.62)	-0.42 (-2.66, 1.81)	-1.82 (-4.15, 0.51)
	-5.12 (-22.51, 12.28)	-1.14 (-13.12, 10.83)	-1.48 (-21.16, 18.19)	-1.12 (-22.25, 20.01)	MA	-0.31 (-2.50, 1.87)	-0.46 (-1.98, 1.07)	-0.24 (-2.13, 1.65)	-1.15 (-3.14, 0.83)
	-7.42 (-22.30, 7.45)	-3.45 (-17.15, 10.26)	-3.79 (-23.89, 16.31)	-3.43 (-24.98, 18.12)	-2.31 (-16.97, 12.36)	AS	-0.77 (-2.39, 0.85)	-0.07 (-2.07, 1.93)	-1.47 (-3.39, 0.46)
	-11.87 (-26.00, 2.27)	-7.89 (-15.98, 0.20)	-8.23 (-24.03, 7.56)	-7.87 (-25.45, 9.71)	-6.75 (-18.47, 4.97)	-4.44 (-16.90, 8.02)	CM	-0.70 (-2.11, 0.71)	-0.70 (-2.25, 0.85)
	-14.85 (-32.73, 3.02)	-10.88 (-21.56, -0.19)	-11.22 (-31.17, 8.74)	-10.86 (-32.26, 10.55)	-9.74 (-22.26, 2.79)	-7.43 (-22.76, 7.90)	-2.99 (-15.20, 9.22)	EA	-1.39 (-2.85, 0.06)
	-16.46 (-33.03, 0.11)	-12.48 (-22.95, -2.01)	-12.82 (-32.02, 6.37)	-12.46 (-33.16, 8.24)	-11.34 (-20.06, -2.62)	-9.03 (-22.08, 4.02)	-4.59 (-15.51, 6.33)	-1.60 (-12.10, 8.89)	SI

Figure 13 Network meta-analysis of SL and AT［MD(95%CI)］

	SDS										
SAS	MB	-10.81 (-28.01, 6.38)	-1.13 (-10.94, 8.68)	-0.31 (-17.60, 16.98)	-4.38 (-16.27, 7.51)	-4.55 (-17.99, 8.89)	-6.58 (-15.33, 2.18)	-1.93 (-14.65, 10.79)	----	-12.21 (-29.12, 4.71)	-11.39 (-22.49, -0.28)
	-2.03 (-15.44, 11.37)	HBO	-11.94 (-28.72, 4.83)	-11.12 (-33.13, 10.88)	-15.19 (-32.41, 2.02)	-15.36 (-33.34, 2.61)	-17.39 (-32.19, -2.59)	-12.74 (-31.29, 5.80)	----	-23.02 (-43.73, -2.31)	-22.20 (-39.62, -4.78)
	-4.45 (-11.84, 2.93)	-2.42 (-15.23, 10.39)	MA	-0.82 (-15.06, 13.43)	-3.25 (-13.40, 6.89)	-3.42 (-16.31, 9.47)	-5.45 (-13.34, 2.45)	-0.80 (-9.28, 7.68)	----	-11.08 (-27.57, 5.41)	-10.26 (-16.33, -4.19)
	-5.75 (-19.14, 7.65)	-3.71 (-20.71, 13.29)	-1.29 (-12.47, 9.88)	ACE	-4.07 (-21.56, 13.41)	-4.24 (-23.45, 14.97)	-6.27 (-22.55, 10.01)	-1.62 (-18.20, 14.96)	----	-11.90 (-33.68, 9.89)	-11.08 (-26.56, 4.41)
	-7.00 (-16.40, 2.39)	-4.97 (-18.48, 8.54)	-2.55 (-10.49, 5.39)	-1.26 (-14.97, 12.45)	rTMS	-0.17 (-13.63, 13.30)	-2.20 (-10.99, 6.60)	-2.45 (-14.81, 9.91)	----	-7.83 (-24.77, 9.11)	-7.00 (-17.26, 3.25)
	-8.08 (-18.49, 2.33)	-6.05 (-20.11, 8.01)	-3.63 (-13.26, 6.00)	-2.34 (-17.09, 12.41)	-1.08 (-11.62, 9.46)	AS	-2.03 (-12.22, 8.17)	-2.62 (-17.74, 12.50)	----	-7.66 (-25.36, 10.05)	-6.84 (-20.56, 6.88)
	-9.97 (-16.70, -3.25)	-7.94 (-19.54, 3.66)	-5.52 (-10.96, -0.08)	-4.23 (-16.66, 8.20)	-2.97 (-9.90, 3.96)	-1.89 (-9.84, 6.06)	CM	-4.65 (-15.81, 6.52)	----	-5.63 (-20.11, 8.85)	-4.81 (-13.99, 4.37)
	-13.48 (-28.21, 1.25)	-11.45 (-29.37, 6.48)	-9.03 (-22.16, 4.10)	-7.74 (-24.98, 9.51)	-6.48 (-20.36, 7.41)	-5.40 (-21.21, 10.41)	-3.51 (-17.18, 10.16)	EA	----	-10.28 (-28.56, 8.01)	-9.45 (-17.31, -1.60)
	-14.47 (-29.10, 0.16)	-12.43 (-30.27, 5.41)	-10.02 (-23.03, 3.00)	-8.72 (-25.88, 8.44)	-7.46 (-21.24, 6.32)	-6.38 (-22.10, 9.33)	-4.49 (-18.05, 9.07)	-0.99 (-16.61, 14.63)	FB	----	----
	-14.68 (-27.90, -1.47)	-12.65 (-28.90, 3.60)	-10.23 (-22.84, 2.38)	-8.94 (-25.79, 7.91)	-7.68 (-21.00, 5.64)	-6.60 (-20.48, 7.28)	-4.71 (-16.09, 6.67)	-1.20 (-18.99, 16.58)	-0.22 (-17.92, 17.48)	Tuina	0.82 (-16.32, 17.96)
	-16.61 (-26.28, -6.93)	-14.57 (-28.64, -0.50)	-12.15 (-19.16, -5.15)	-10.86 (-24.05, 2.33)	-9.60 (-17.93, -1.27)	-8.52 (-19.78, 2.73)	-6.63 (-14.60, 1.34)	-3.12 (-14.23, 7.99)	-2.14 (-13.12, 8.84)	-1.92 (-15.81, 11.97)	SI

Figure 14 Network meta-analysis of SAS and SDS［MD(95%CI)］

	DA									
**5-HT**	**ACE**	----	----	----	----	----	----	----	----	----
	19.66 (-68.68, 108.00)	**rTMS**	3.29 (-18.06, 24.63)	3.44 (-9.59, 16.48)	**28.96 (13.97, 43.95)**	**75.66 (60.36, 90.96)**	----	**86.05 (61.47, 110.63)**	**90.48 (79.69, 101.27)**	**98.32 (90.76, 105.89)**
	22.28 (-79.05, 123.62)	2.62 (-65.32, 70.56)	**Tuina**	6.73 (-18.15, 31.61)	**32.25 (11.10, 53.40)**	**78.95 (57.53, 100.36)**	----	**89.33 (58.24, 120.43)**	**93.77 (75.36, 112.18**	**101.61 (79.10, 124.12)**
	31.53 (-71.59, 134.66)	11.87 (-73.59, 97.34)	9.25 (-89.59, 108.08)	**EA**	**25.52 (5.82, 45.23)**	**72.22 (52.43, 92.01)**	----	**82.61 (56.15, 109.07)**	**87.04 (70.31, 103.78**	**94.88 (84.26, 105.50)**
	37.30 (-37.31, 111.91)	17.64 (-29.66, 64.95)	15.02 (-53.55, 83.59)	5.77 (-65.42, 76.96)	**MA**	**46.70 (31.60, 61.80)**	----	**57.08 (29.95, 84.22)**	**61.52 (51.11, 71.93)**	**69.36 (52.75, 85.97)**
	46.38 (-52.69, 145.46)	26.72 (-36.81, 90.26)	24.10 (-56.67, 104.87)	14.85 (-81.67, 111.38)	9.08 (-56.12, 74.28)	**FB**	----	10.39 (-15.14, 35.92)	**14.82 (3.88, 25.76)**	**22.66 (5.94, 39.38)**
	46.27 (-45.94, 138.48)	26.61 (-26.78, 80.00)	23.98 (-46.97, 94.94)	14.74 (-74.72, 104.19)	8.96 (-45.22, 63.15)	-0.12 (-69.11, 68.88)	**MB**	----	----	----
	63.83 (-30.29, 157.95)	44.17 (-9.98, 98.31)	41.54 (-34.42, 117.51)	32.30 (-59.13, 123.72)	26.52 (-30.86, 83.91)	17.44 (-39.98, 74.86)	17.56 (-45.77, 80.89)	**AS**	4.44 (-20.62, 29.49)	12.27 (-11.96, 36.51)
	**89.44 (6.56, 172.31)**	**69.78 (34.90, 104.65)**	**67.15 (8.85, 125.46)**	57.91 (-21.90, 137.71)	**52.13 (16.05, 88.22)**	43.05 (-12.84, 98.94)	**43.17 (2.74, 83.60)**	25.61 (-23.09, 74.31)	**CM**	7.84 (-5.11, 20.78)
	**122.60 (35.07, 210.12)**	**102.94 (63.99, 141.89)**	**100.31 (30.36, 170.27**	**91.07 (6.44, 175.69)**	**85.29 (39.53, 131.06**	**76.21 (14.10, 138.33)**	**76.33 (20.40, 132.26**	**58.77 (11.64, 105.90)**	33.16 (-5.50, 71.82)	**SI**

Figure 15 Network meta-analysis of 5-HT and DA［MD(95%CI)］

**NE**	EA							
	-1.46 (-21.37, 18.46)	rTMS						
	-2.77 (-29.11, 23.57)	-1.32 (-18.64, 16.01)	AS					
	-3.90 (-18.43, 10.63)	-2.44 (-16.06, 11.18)	-1.12 (-23.09, 20.85)	MA				
	-1.96 (-65.04, 61.13)	-0.50 (-61.94, 60.94)	0.82 (-62.99, 64.63)	1.94 (-59.45, 63.33)	Tuina			
	-4.16 (-37.81, 29.50)	-2.70 (-33.16, 27.76)	-1.38 (-36.38, 33.62)	-0.26 (-30.62, 30.10)	-2.20 (-69.36, 64.96)	MB		
	-15.56 (-32.90, 1.78)	**-14.10 (-23.90, -4.30)**	-12.78 (-32.61, 7.04)	**-11.66 (-21.13, -2.19)**	-13.60 (-74.25, 47.05)	-11.40 (-40.25, 17.45)	CM	
	-20.00 (-42.30, 2.29)	**-18.55 (-28.71, -8.38)**	**-17.23 (-31.26, -3.20)**	-16.10 (-33.02, 0.81)	-18.04 (-80.30, 44.21)	-15.84 (-47.91, 16.22)	-4.44 (-18.45, 9.56)	SI

Figure 16 Network meta-analysis of NE［MD(95%CI)］

Funnel plot

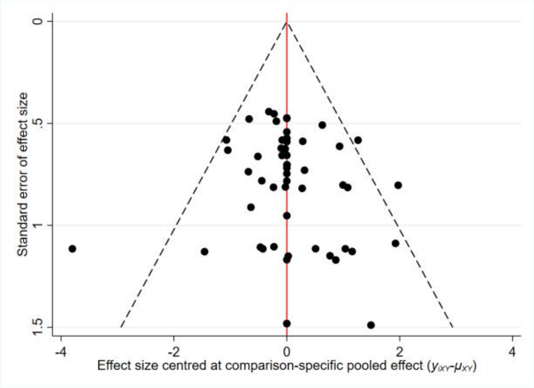

Figure 17 Funnel plot of total effective rate

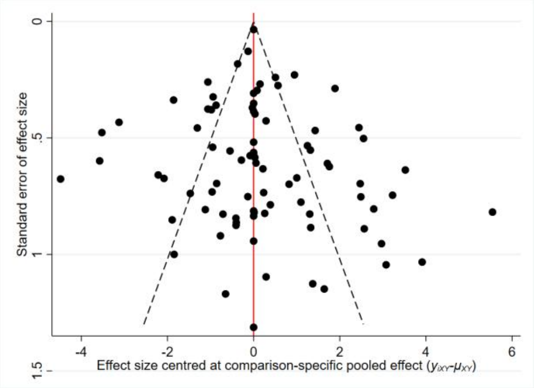

Figure 18 Funnel plot of PSQI

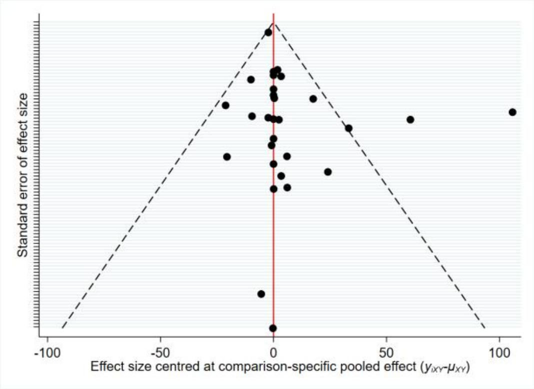

Figure 19 Funnel plot of TST

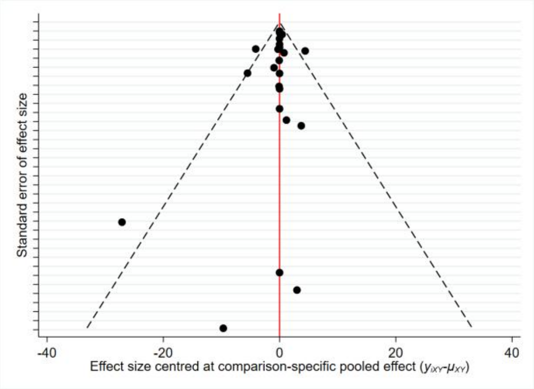

Figure 20 Funnel plot of SL

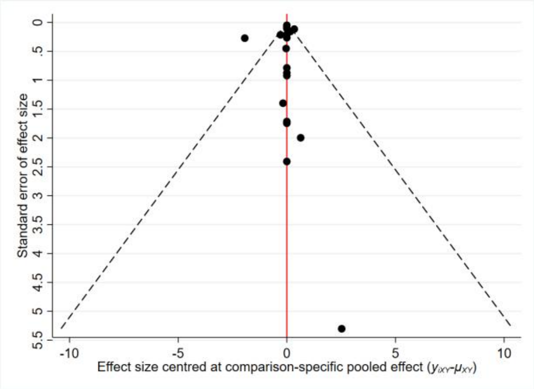

Figure 21 Funnel plot of AT

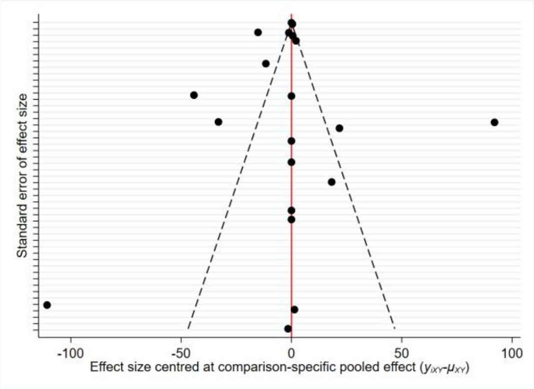

Figure 22 Funnel plot of 5-HT

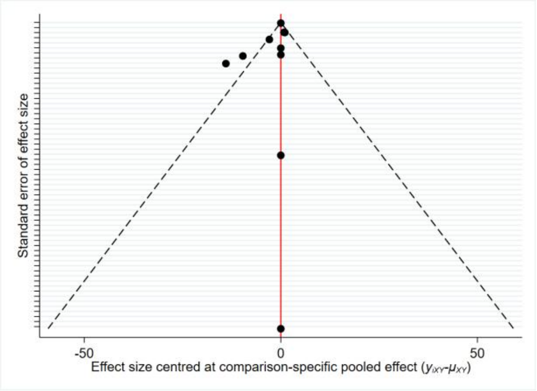

Figure 23 Funnel plot of NE

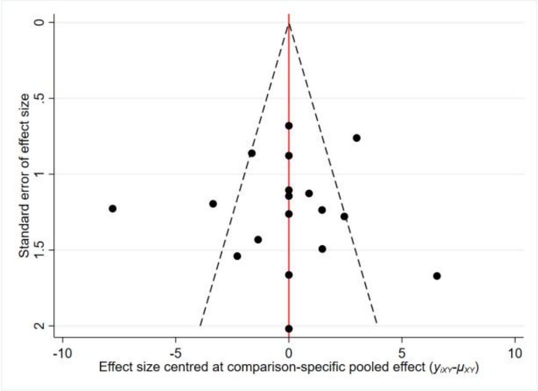

Figure 24 Funnel plot of SAS

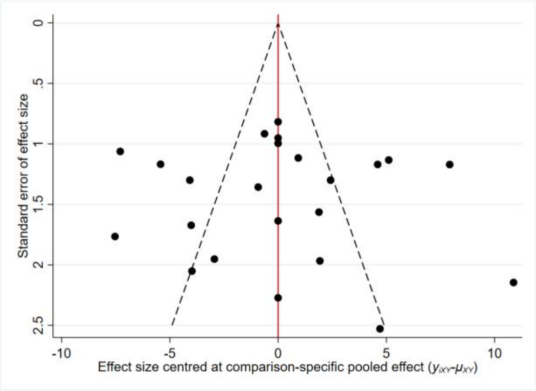

Figure 25 Funnel plot of SDS

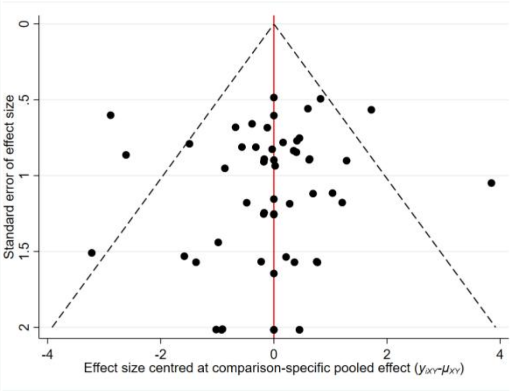

Figure 26 Funnel plot of adverse reaction

Table 13 Summary of results of adverse events for all interventions

Intervention	Type of adverse reaction					
	Pain	Dizziness	Hematoma	drowsiness	thirst	Other
ACE	4	-	3	-	-	Nodule(2); Fever(1); Allergy(2)
rTMS	19	5	-	-	1	Sweatiness(1)
Tuina	-	2	-	2	2	-
HBO	4	-	-	-	-	Tinnitus(6)
EA	10	1	1	-	-	Numbness(1); Bleeding(1); dyspepsia(6); pruritus(1)
MB	-	2	1	2	2	Blurred vision(1); Bleeding(1); constipation(1); empyrosis(1)
FB	-	-	-	2	2	Nausea(1)
MA	10	8	11	-	-	Halo stitches(2); Numbness(1); palpitation(1); Bleeding(3); Nausea(1)
AS	9	1	1	-	-	Palpitation(1)
CM	14	15	-	20	26	Fatigue(11); Blurred vision(9): Constipation(1); Nausea(4)
SI	6	8	1	-	-	Numbness(1); Fatigue(1)
